# Characterizing viral within-host diversity in fast and non-equilibrium demo-genetic dynamics

**DOI:** 10.3389/fmicb.2022.983938

**Published:** 2022-10-05

**Authors:** Maryam Alamil, Gaël Thébaud, Karine Berthier, Samuel Soubeyrand

**Affiliations:** ^1^INRAE, BioSP, Avignon, France; ^2^Department of Mathematics and Computer Science, Alfaisal University, Riyadh, Saudi Arabia; ^3^PHIM Plant Health Institute, INRAE, Univ Montpellier, CIRAD, Institut Agro, IRD, Montpellier, France; ^4^INRAE, Pathologie Végétale, Montfavet, France

**Keywords:** diversity indices, genome evolution, kinetic model, simulation model, virus evolution, within-host pathogen diversity

## Abstract

High-throughput sequencing has opened the route for a deep assessment of within-host genetic diversity that can be used, e.g., to characterize microbial communities and to infer transmission links in infectious disease outbreaks. The performance of such characterizations and inferences cannot be analytically assessed in general and are often grounded on computer-intensive evaluations. Then, being able to simulate within-host genetic diversity across time under various demo-genetic assumptions is paramount to assess the performance of the approaches of interest. In this context, we built an original model that can be simulated to investigate the temporal evolution of genotypes and their frequencies under various demo-genetic assumptions. The model describes the growth and the mutation of genotypes at the nucleotide resolution conditional on an overall within-host viral kinetics, and can be tuned to generate fast non-equilibrium demo-genetic dynamics. We ran simulations of this model and computed classic diversity indices to characterize the temporal variation of within-host genetic diversity (from high-throughput amplicon sequences) of virus populations under three demographic kinetic models of viral infection. Our results highlight how demographic (viral load) and genetic (mutation, selection, or drift) factors drive variations in within-host diversity during the course of an infection. In particular, we observed a non-monotonic relationship between pathogen population size and genetic diversity, and a reduction of the impact of mutation on diversity when a non-specific host immune response is activated. The large variation in the diversity patterns generated in our simulations suggests that the underlying model provides a flexible basis to produce very diverse demo-genetic scenarios and test, for instance, methods for the inference of transmission links during outbreaks.

## 1. Introduction

RNA viruses, such as Influenza A, Ebola, and Hepatitis C viruses, are often referred as fast evolving pathogens because of their high mutation rates and rapid generation time (Biek et al., 2015; Nelson and Hughes, 2015; Picard et al., 2017). These characteristics hold at the multi-host level as well as at the within-host level. The development of sequencing technologies has contributed to unravel the level of genetic diversity within a single host and how it varies spatially and temporally during the course of the infection, due to mutation, selection, and genetic drift processes acting at the within-host scale (Pybus and Rambaut, 2009; Alizon et al., 2011; Gutiérrez et al., 2012; Simmons et al., 2012; Abel et al., 2015; Cuevas et al., 2015; Nelson and Hughes, 2015; Poirier and Vignuzzi, 2017). Typically, a deep assessment of temporal changes in within-host genetic diversity can be achieved using whole genome high-throughput sequencing (HTS) approaches on serial samples from infected hosts. However, and although most RNA viruses have relatively small genome sizes, accurate whole genome sequencing of numerous samples still remains costly and time consuming (Kulkarni and Frommolt, 2017). Alternatively, within-host genetic diversity can be approached by high-throughput amplicon sequencing (HTAS) techniques, which can be used to identify distinct genotypes for a target marker of a few hundred bases length within the host while genotyping a high number of samples through ad hoc multiplexing techniques (Galan et al., 2010, 2012; Piry et al., 2017). Such techniques are less costly and produce data that can be easily handled and analyzed with limited computational resources and bioinformatics, e.g., using the R package dada2 (Callahan et al., 2016).

Within-host genetic diversity of viruses is of particular interest for inferring (potentially indirect) epidemiological links between hosts and even reconstructing transmission chains in outbreaks. Before the use of within-host genetic diversity for such inferences, one essentially exploited the high mutation rate and rapid generation time of viruses (Brunker et al., 2012; Picard et al., 2017). Typically, these approaches used genetic-space-time relations at the multi-host level to reconstruct transmission links during outbreaks (Cottam et al., 2008; Morelli et al., 2012; Ypma et al., 2012, 2013; Jombart et al., 2014; Mollentze et al., 2014; Hall et al., 2015; Lau et al., 2015; Valdazo-González et al., 2015). In most of the earliest approaches that have been developed, the host unit was (implicitly) considered as a homogeneous environment, within which the viral population at a given time was represented by a unique sequence, such as the consensus sequence or the majority sequence.

However, recent approaches have exploited within-host genetic diversity and the degree of genetic similarity (in a broad sense) between viral genotypes collected from different hosts for transmission chain reconstruction (Hughes et al., 2012; Morelli et al., 2012; Murcia et al., 2012; Walker et al., 2013; Didelot et al., 2014; Jombart et al., 2014; Worby et al., 2014; De Maio et al., 2018; Leitner and Romero-Severson, 2018; Wymant et al., 2018; Alamil et al., 2019). To evaluate the performance of these approaches in numerous diverse and challenging settings, we need simulation models of viral within-host genetic diversity and tools to characterize this diversity. Here, we propose such a framework, based on the work of Worby and Read (2015) on the simulation of evolutionary and epidemiological dynamics, as well as classical viral kinetic models and widely used diversity indices. This framework was designed to possibly generate non-equilibrium fast evolutionary dynamics. Briefly, the “non-equilibrium” feature means that the system can bifurcate into new dynamic steady states (Chaisson, 2004), and the adjective “fast” indicates that such bifurcations may arise quite frequently. In the context of virology considered here, a “frequent bifurcation” is typically manifested by a change in the dominant viral genotype during the infection of a host, as observed, e.g., by Hughes et al. (2012). Multiple mechanisms related to selection and drift can drive such changes and our approach is to account for them implicitly by going beyond the binomial or multinomial draws intuitively applied to modeling genotype replication.

In our approach, the within-host virus population is simulated by generating genotypes (i.e., sequence fragments) and their proportions conditional on a demographic kinetics to be specified. The resulting computer-based demo-genetic dynamics can be generated under numerous conditions and can be monitored like in real situations using HTAS longitudinal samples (i.e., samples collected from a unique host at different time points during the infection). In the model, demographic effects are essentially represented by a founder effect (i.e., the set of genotypes initiating the infection), which may be relatively strong (Abel et al., 2015; Poirier and Vignuzzi, 2017), and a demographic kinetics described by a set of differential equations and quantifying the variation of the viral load during the course of the infection, which is represented by a set of differential equations any other mathematical formalism may be used for the demographic kinetics as soon as it provides a quantity of virions across time; e.g., see Yuan and Allen (2011), for models based on stochastic differential equations and continuous-time Markov chains. We consider three examples of kinetic models, all including a latent period, and respectively, representing an acute infection, a persistent infection and an infection mitigated by an immune response. These examples were chosen more for their ability to produce contrasting viral load dynamics than for their applicability to a specific case study.

Genetic effects incorporated into the model correspond to the mutation and replication processes. Nucleotide substitutions are assumed to occur randomly at a constant rate. Mutation effects are handled by classifying substitutions into lethal (leading to extreme negative selection) and non-lethal. Genotype replication is simulated by successive over-dispersed multinomial draws with a size equal to the current quantity of virions that is governed by the chosen kinetic model. The replication success represents the relative fitness of the genotypes, which can vary during the course of the infection *via* the over-dispersion of the multinomial draws. This over-dispersion is governed by a shuffling process noising the current vector of genotype proportions. When this process is applied, a rare genotype at generation *t* can significantly increase in proportion at generation *t* + 1. This process implicitly mimics positive selection, genetic drift and spatio-temporal variation in genotype multiplication (occurring, e.g., when a genotype invades a new part of the host that is more favorable). Thus, overall, the stochastic model that we propose implicitly or explicitly encompasses several biological mechanisms such as natural selection and genetic drift and produces fast and non-equilibrium demo-genetic dynamics.

The model briefly described above was designed for the evaluation, in diverse and challenging demo-genetic situations, of the performance of methods that reconstruct transmission trees by exploiting within-host genetic diversity data. However, we focus in this article on the characterization of the genetic diversity resulting from this simulation model. Thus, in what follows, we propose a comprehensive mathematical description of the model and we investigate the influence of the parameters on temporal variations in genetic diversity. This investigation is performed using several diversity indices, and contributes to a better understanding of the main drivers of within-host genetic evolution and pathogen population divergence. The results especially highlight the major impact of the shuffling process, the non-monotonic relationship between pathogen population size and genetic diversity, and the reduction of the impact of mutation on diversity when a host immune response is activated. These elements are discussed in the last section of this article. An R code called MoWPP (Model of Within-host Pathogen Population dynamics) allowing the user to run the model and compute the diversity indices is provided at https://doi.org/10.5281/zenodo.6783246.

## 2. Materials and methods

The following three subsections detail the modeling framework using the mathematical formalism. The last subsection provides a concise overview of the model *via* an algorithmic description.

### 2.1. Kinetic models

We consider that the size of the within-host pathogen population varies over time. To quantify this temporal variation, one can use a wide range of kinetic models that were developed to study within-host dynamics of many pathogens (Perelson and Nelson, 1999; Nowak and May, 2000; Baccam et al., 2006; Beauchemin et al., 2008; Handel et al., 2010; Saenz et al., 2010; Beauchemin and Handel, 2011; Smith and Perelson, 2011; Pawelek et al., 2012; Canini and Perelson, 2014; Hernandez-Vargas, 2019), including SARS-CoV-2 (Du and Yuan, 2020; Gonçalves et al., 2020; Goyal et al., 2020; Hernandez-Vargas and Velasco-Hernandez, 2020; Pinky and Dobrovolny, 2020; Wang et al., 2020; Blanco-Rodriguez et al., 2021; Ghosh, 2021). These models are grounded on sets of ordinary differential equations (ODE) basically governing the numbers of susceptible target cells, infected cells, and virions.

For this article primarily focused on a new modeling framework coupling viral kinetics and micro-evolution, we considered three kinetic models that were chosen because they generate clearly contrasting viral-load patterns (with one peak, one plateau, and two peaks), but many other kinetic models may be considered (see Section 4.2). The three models include a latent period modeled by considering two compartments of infected cells: those not producing virions yet and those actively producing virions. The first model corresponds to acute infections (Baccam et al., 2006) and has been recently used to describe the within-host kinetics of SARS-CoV-2 with or without latent period (Abuin et al., 2020; Hernandez-Vargas and Velasco-Hernandez, 2020). The second model is a direct derivation of the first model, that allows us to transform the acute infection model into a persistent infection model (i.e., presenting a plateau) over the 10-day study period by setting the death rate of infectious cells at zero (it is viewed as a toy model). The third model is a hybrid between the model of Baccam et al. (2006) (allowing a latent period in virion production) and the model of Pawelek et al. (2012) introducing an immune response.

In this section, the state variables (*S*, *I*, *V*, …) are functions of continuous-time *t* ≥ 0.

#### 2.1.1. Acute and persistent infection models

The acute infection model is derived from a simple viral kinetic model describing the dynamics between susceptible target cells (S), infected cells (I), and virions (V) (Baccam et al., 2006; Beauchemin and Handel, 2011). It illustrates the eclipse phase dynamics. The eclipse phase is the time span between the entry of the virus into the target cells and the release of the virions produced by these newly infected cells. The delay in the viral production is modeled by defining two separate populations of infected cells: the infected population that is not yet producing virions, *I*_1_, and the infectious population that is actively producing virions, *I*_2_. The following set of differential equations (Baccam et al., 2006; Beauchemin and Handel, 2011) defines the acute infection model:


(1) $$\begin{array}{l} \{\begin{array}{l} \frac{dS}{dt}=-\beta SV\\ \frac{dI_{1}}{dt}=\beta SV-kI_{1}\\ \frac{dI_{2}}{dt}=kI_{1}-\delta I_{2}\\ \frac{dV}{dt}=pI_{2}-cV \end{array} \end{array}$$

where the susceptible cells, *S*, are converted at rate β into infected cells, *I*_1_, upon interaction with virions, *V*. Infected cells become infectious at rate *k*; in other words, 1/*k* is the average transition time from *I*_1_ to *I*_2_. The virions, *V*, are assumed to be produced at rate *p* and cleared at rate *c*.

To model a persistent infection (over a relatively short-time period, i.e., 10 days in our simulation study), we use the acute model of Equation (1) and we set the death rate δ of infectious cells *I*_2_ to zero. In that respect, we assume that the infectious cells *I*_2_ are not removed. This corresponds to both the absence of cytotoxic effects of the virus and a delay/lack of activation of the immune response against the infectious cells, resulting in negligible damage to these cells (Boldogh et al., 1996), at least over the short time period considered in our work.

**Figure 1:**
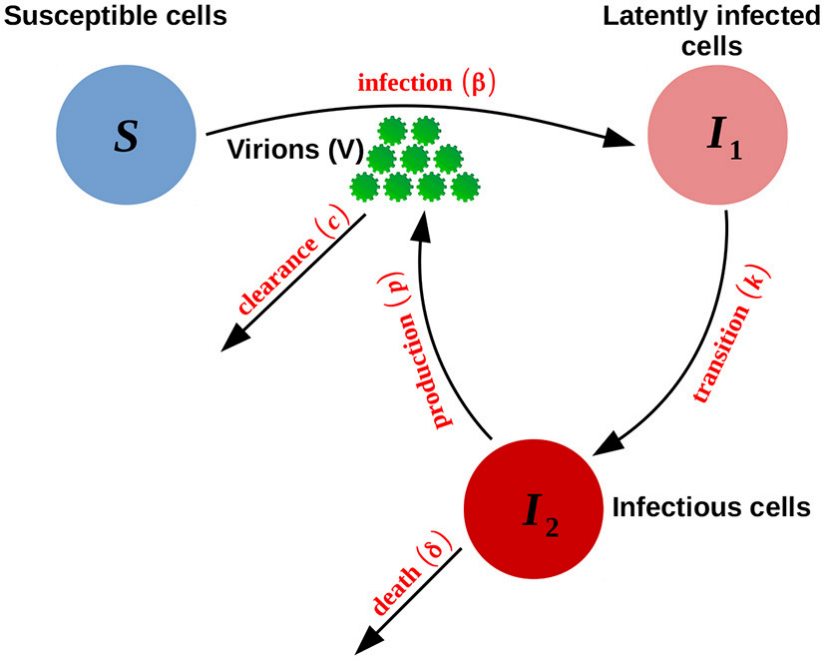
Schematic representation of the acute and persistent models defined by Equation (1). The persistent model is obtained by setting the death rate of infectious cells δ to zero.

A schematic diagram of these acute and persistent infection models is shown in Figure 1.

#### 2.1.2. Model with immune system reactions

**Figure 2:**
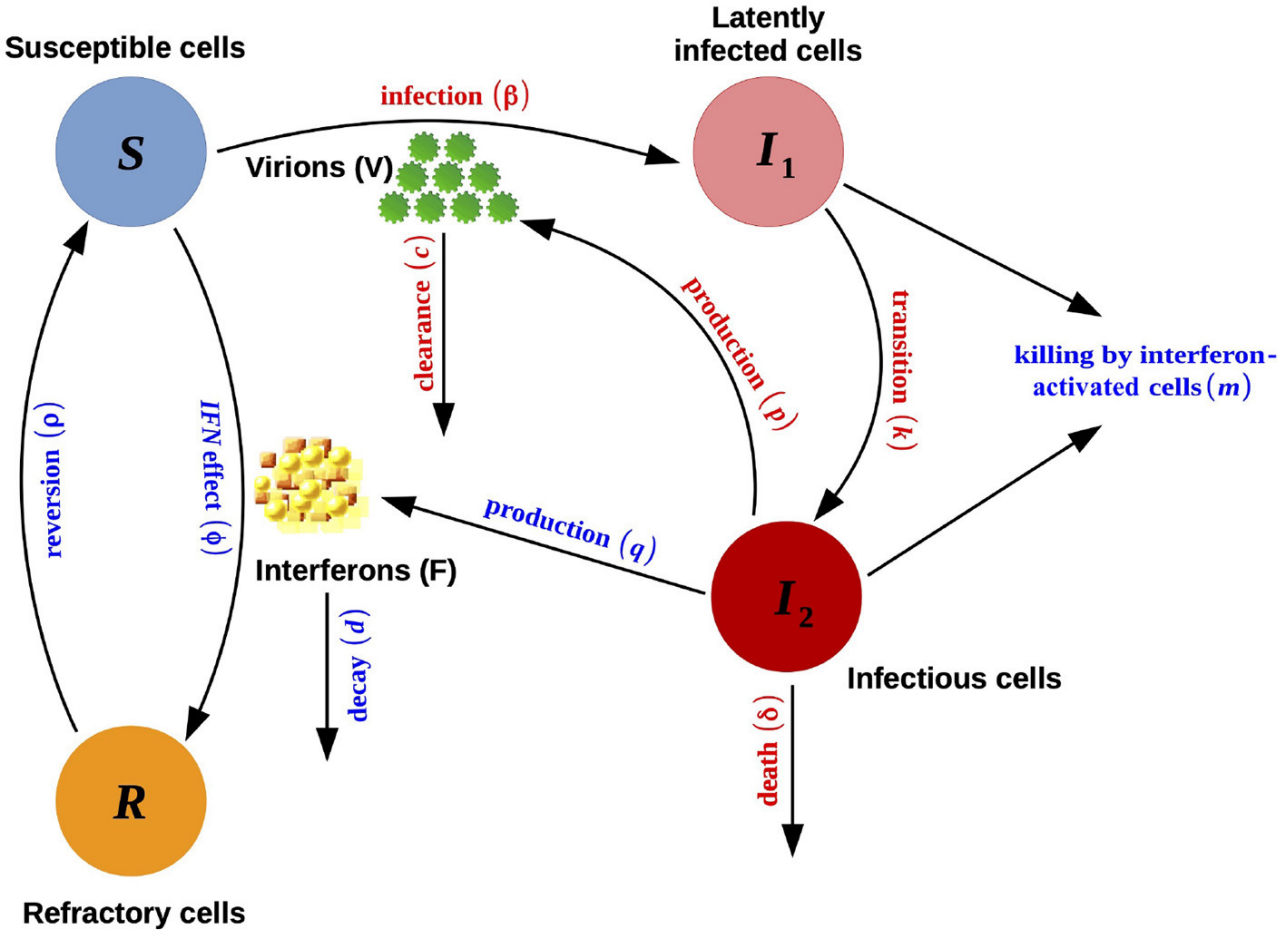
Schematic representation of the model with immune system reaction defined in Equation (2). This scheme is an edited version (with permission) of the original scheme presented by Pawelek et al. (2012).

A third model accounts for an immune response. Innate immunity through interferon (IFN) induction is modeled by adding two compartments to the acute-infection model defined by Equation (1): the IFNs (*F*) and the refractory uninfected cells (*R*). The rising adaptive immune response is modeled as an increase in the death rate of the infectious cells, δ, after an initial delay. This model, illustrated in Figure 2, is defined by:


(2) $$\begin{array}{l} \{\begin{array}{l} \frac{dS}{dt}=-\beta SV-\phi SF+\rho R\\ \frac{dI_{1}}{dt}=\beta SV-kI_{1}-mI_{1}F\\ \frac{dI_{2}}{dt}=kI_{1}-\delta I_{2}-mI_{2}F\\ \frac{dR}{dt}=\phi SF-\rho R\\ \frac{dV}{dt}=pI_{2}-cV\\ \frac{dF}{dt}=qI_{2}-dF \end{array} \end{array}$$

where IFNs are secreted only by infectious cells *I*_2_ at rate *q* and decay at rate *d*; upon exposure to these signaling proteins, all infected cells incur an (additional) death rate *m*, and susceptible cells become refractory to infection at rate ϕ (refractory cells revert to the susceptible state at rate ρ); δ is defined as follows:


$$\begin{array}{l} \delta =\{\begin{array}{ll} \delta_{\text{I}} & \text{if }t<t_{\text{I}}\\ \delta_{\text{I}}e^{\sigma_{\text{I}}\left(t-t_{\text{I}}\right)} & \text{otherwise} \end{array} \end{array}$$

with 1/δ_I_ the mean lifespan of the infectious cells before the rise of the immune response, and σ_I_ the speed at which the death rate increases after the time *t*_I_ when the adaptive immune response starts (Pawelek et al., 2012).

#### 2.1.3. Kinetic parameter values and model solving

Values of parameters and initial values of variables used thereafter for simulating changes in the viral load during 10 days are provided in Tables 1, 2 for the three kinetic models. Many of these values are taken from previous studies (Baccam et al., 2006; Pawelek et al., 2012), in which parameters were either fixed by the authors or estimated with a least square approach between the kinetic model and experimental data collected from patients infected by H1N1 (Baccam et al., 2006) or from unvaccinated ponies infected by EIV (Pawelek et al., 2012).

**Table 1:** Description and values of variables and parameters used in the acute and persistent infection models described by Equation (1).

**Symbol**	**Definition**	**Unit**	**Value**
*S*	Uninfected cells that are susceptible to infection	Cells	Initial value: 4 × 10^8^
*I*_1_	Infected cells not producing virus	Cells	Initial value: 0
*I*_2_	Infected cells actively producing virus	Cells	Initial value: 0
*V*	Viral load	TCID_50_/ml	Initial value: 4.9
β	Rate of susceptible target cell infection	(TCID_50_/ml)^−1^.d^−1^	5.3 × 10^−6^
*k*	1/*k*: average transition time from *I*_1_ to *I*_2_	d^−1^	4
δ	Death rate of infected cells *I*_2_ That actively produce virus	d^−1^	3.8 | 0^*^
*p*	Viral production rate	(TCID_50_/ml).d^−1^	0.05 | 0.0095^*^
*c*	Clearance rate of virions	d^−1^	3.8

* First value for the acute infection model, second value for the persistent infection model.

In the acute infection model, initial values of S, *I*_1_, *I*_2_, and *V* and values of β, *k*, δ, and *c* are those obtained for patient 4 in Baccam et al. (2006, Table 3). The same values were used for the persistent infection model, except δ that was set to zero. In both models, *p* was chosen such that the maximum viral load over the 10-days study period is *V*_max_ = 10^6^ virions.

d stands for day in the unit column.

**Table 2:** Description and values of variables and parameters used in the model with immune response described by Equation (2).

**Symbol**	**Definition**	**Unit**	**Value**
*S*	Uninfected cells that are susceptible to infection	Cells	Initial value: 3.5 × 10^11^
*I*_1_	Infected cells not producing virus	Cells	Initial value: 0
*I*_2_	Infected cells actively producing virus	Cells	Initial value: 0
*R*	Uninfected refractory cells	Cells	Initial value: 0
*F*	Interferon	IFN fold change	Initial value: 5.3
*V*	Viral load	TCID_50_/ml	Initial value: 3.5 × 10^−1^
β	Rate of susceptible target cell infection	(TCID_50_/ml)^−1^.d^−1^	8.3 × 10^−6^
ϕ	Rate of the IFN-induced antiviral efficacy	(IFN fold change)^−1^.d^−1^	9 × 10^−4^
ρ	Reversion rate from refractory to susceptible state	d^−1^	1.5
*k*	1/*k*: average transition time from *I*_1_ to *I*_2_	d^−1^	0.55
δ_I_	Death rate of infected cells before onset of the adaptive immune response	d^−1^	4
*t*_I_	Time at which the adaptive immune response starts	d	6
σ_I_	Speed of the death rate increase		4
*m*	Killing rate of infected cells by IFN-activated NK cells	(IFN fold change)^−1^.d^−1^	2.9 × 10^−3^
*p*	Viral production rate	(TCID_50_/ml).d^−1^	4.8 × 10^−3^
*c*	Clearance rate of virions	d^−1^	11.5
*q*	Rate of IFN production	(IFN fold change).cell^−1^	1.1 × 10^−5^
d	Rate of IFN decay	d^−1^	0.72

Initial values of the number of susceptible, infected and refractory cells and the value of β were taken from Pawelek et al. (2012, Tables 1, 2). The other values were chosen such that the viral load has two clearly distinct peaks over 10 days.

d stands for day in the unit column.

The viral production rate, *p*, is chosen such that the maximum viral load reached during the infection period, say *V*_max_, is the same for the three different models (we use $V_{\text{max}}=10^{6}$ virions). For each model, parameter *p* is computed by minimizing (with respect to *p*) the squared difference, $\Delta_{p}={\left(V_{\text{max}}-{\bar{V}}_{p}\right)}^{2}$, between *V*_max_ and the maximum value ${\bar{V}}_{p}$ (over a 10-day time period) of the number of virions *V* obtained by solving the system of ODEs.

The systems of ODEs can be numerically solved with the ode function of the deSolve package in R. We used a 0.001 day resolution in the applications for defining the time sequence for which values of states variables (*S*, *I*, *V*, …) were computed.

### 2.2. Demo-genetic model with fast variation

To generate within-host genetic diversity of a pathogen population with a non-equilibrium fast evolutionary dynamics, we build a discrete-time stochastic model simulating genotypes and their frequencies at each generation during an infection period. Numerous data sets can be generated with this model under various demo-genetic situations that can lead to fast-evolving dynamics and consequently to significant changes in the viral composition.

Our model integrates several demographic and genetic factors, namely the kinetic model, the growth of genotypes, the mutations of genomes and two fitness components, namely the shuffling process and the elimination of lethal genome. In what follows, we first describe the growth and mutation stages that form the skeleton of the demo-genetic model (Sections 2.2.1, 2.2.2). Then, we present the shuffling process in Section 2.2.3, which is an “option” in our model for noising the proportions of genotypes during the growth stage (it can be used for favoring the growth of minor variants, for example, and hence favoring “frequent bifurcations”). Finally, we present the elimination of lethal genomes, which is also an “option” in our model that can be applied after the mutation stage.

In the model, the sum of genotype frequencies at each generation (i.e., the pathogen population size) is assumed to be the quantity of virions, *V*, given by a viral kinetic model such as those presented in Section 2.1. We only need values of *V* at the discrete times corresponding to the generations. Thereafter, the generation and the day coincide (in the literature, the reproductive cycle of viruses ranges from 8 to more than 72 h; Roizman, 1996); time *t* takes integer values corresponding to the generation and coinciding with integer values of time in the definition of the kinetic models provided in Section 2.1.

Host infection is initiated by the introduction of a single genotype defined by a nucleotide sequence of length *L*, each nucleotide being uniformly drawn among {A,C,G,T}. At any time *t* (i.e., generation) during the infection period, the within-host pathogen population is represented by a set of *n*(*t*) different genotypes *G*(*t*) = {*g*_1_(*t*), ..., *g*_*n*(*t*)_(*t*)} and their absolute frequencies *F*(*t*) = {*f*_1_(*t*), ..., *f*_*n*(*t*)_(*t*)} (*g*_*i*_(*t*) is the *i*-th genotype at time *t*; *f*_*i*_(*t*) is the frequency of the *i*-th genotype at time *t*). Below, to complement the definition of the stochastic demo-genetic model, we describe how {*G*(*t*), *F*(*t*)} are generated by a sequential procedure, conditionally on {*G*(*t* − 1), *F*(*t* − 1)} and *V*(*t*).

#### 2.2.1. Growth

At each time *t*, genotypes undergo a growth stage constrained by the fact that the total quantity of genomes goes from $V\left(t-1\right)=\overset{n\left(t-1\right)}{\underset{i=1}{\sum }}f_{i}\left(t-1\right)$ to *V*(*t*). This stage is performed with a conditional multinomial draw with size *V*(*t*) and probabilities P^*^(*t* − 1) equal to standardized noisy versions of the proportions $P\left(t-1\right)=\frac{1}{V\left(t-1\right)}F\left(t-1\right)$ of the genotypes in the set *G*(*t* − 1) (Section 2.2.3 specifies P^*^):


(3) $$\begin{array}{l} F^{\prime }\left(t\right)|P^{*}\left(t-1\right),V\left(t\right)\sim \text{Multinomial }\left(V\left(t\right)P^{*}\left(t-1\right)\right) \end{array}$$

where $F^{\prime }\left(t\right)=\left\{f_{1}^{\prime }\left(t\right),...,f_{n\left(t-1\right)}^{\prime }\left(t\right)\right\}$ is the frequency vector, after the growth stage, of the *n*(*t* − 1) genotypes constituting the *G*(*t* − 1) family.

After the growth stage and before the mutation stage, all genotypes with zero-frequencies are removed. Hence, we introduce:


$$\begin{array}{l} G^{*}\left(t\right)=\left\{g_{i}\left(t-1\right)\;:\;i=1,\ldots ,n\left(t-1\right),f_{i}^{\prime }\left(t\right)>0\right\}\subset G\left(t-1\right)\\ \;=\left\{g_{1}^{*}\left(t\right),...,g_{m\left(t\right)}^{*}\left(t\right)\right\} \end{array}$$

the set of non-zero frequency genotypes (*m*(*t*) ≤ *n*(*t* − 1) is the number of these genotypes), and $F^{*}\left(t\right)=\left\{f_{1}^{*}\left(t\right),...,f_{m\left(t\right)}^{*}\left(t\right)\right\}$ the vector of the corresponding frequencies [F^*^(*t*) is obtained by removing the null elements of the vector *F*′(*t*)].

#### 2.2.2. Mutations

After the growth of genotypes and the removal of those with zero-frequencies, genomes undergo a mutation stage. At this stage, the number *N*_*v*_(*t*) of mutations occurring in the genome *v* ∈ {1, …, *V*(*t*)}, whose genotype is denoted by ${ɤ}_{v}\in G^{*}\left(t\right)$, follows a binomial distribution with size *L* (which is the genome length) and probability μ (which is the mutation rate per nucleotide per generation):


$$\begin{array}{l} N_{v}\left(t\right)\underset{\text{indep.}}{\sim }\text{Binomial}\left(L,\mu \right),\;\forall v\in \left\{1,\ldots ,V\left(t\right)\right\}. \end{array}$$

Let $V\left(t\right)=\left\{v=1,\ldots ,V\left(t\right):N_{v}\left(t\right)>0\right\}$ denote the set of genomes undergoing at least one mutation. For each v∈V(t), *N*_*v*_(*t*) indices, noted *j*_1_, …, *j*_*N*_*v*_(*t*)_, are selected uniformly with replacement from {1, …, *L*} (drawing mutated positions with replacement allows us to take into account multiple mutations on the same nucleotide site, which means that the effective mutation rate is slightly lower than μ; note however that given the parameter values that we use in the results section, this event is extremely rare). Then, for *j* from *j*_1_ to *j*_*N*_*v*_(*t*)_, the *j*-th nucleotide ɤ_*v*_(*j*) of genome *v* whose genotype is written ɤ_*v*_ = {ɤ_*v*_(1), …, ɤ_*v*_(*L*)} is updated by randomly and uniformly drawing a new nucleotide from the set {A,C,G,T}, excluding the current value of ɤ_*v*_(*j*). Let ${\tilde{ɤ}}_{v}$ denote the genotype obtained using this iterative procedure.

Let $\overset{\sim }{V}\left(t\right)$ designate the set of genomes in V(t) which remain after the elimination of possible lethal genomes [see Section 2.2.4; $\overset{\sim }{V}\left(t\right)=V\left(t\right)$ if none of the genomes are lethal]. Assigning (in an arbitrary order) the indices *m*(*t*) + 1, …, *m*(*t*) + *q*(*t*) to these *q*(*t*) genotypes [where *q*(*t*) is the length of $\overset{\sim }{V}\left(t\right)$], noting $\left\{{\tilde{g}}_{m\left(t\right)+1}\left(t\right),\ldots ,{\tilde{g}}_{m\left(t\right)+q\left(t\right)}\left(t\right)\right\}=\left\{{\tilde{ɤ}}_{v}:v\in \overset{\sim }{V}\left(t\right)\right\}$ and ${\tilde{g}}_{i}\left(t\right)=g_{i}^{*}\left(t\right)$ for each *i* ∈ {1, …, *m*(*t*)}, the genotype set is henceforth:


$$\begin{array}{l} \tilde{G}\left(t\right)=G^{*}\left(t\right)\cup \left\{{\tilde{ɤ}}_{v}\;:\;v\in \overset{\sim }{V}\left(t\right)\right\}\\ \;=\left\{{\tilde{g}}_{1}\left(t\right),...,{\tilde{g}}_{m\left(t\right)+q\left(t\right)}\left(t\right)\right\}. \end{array}$$

In that respect, the set of frequencies corresponding to the genotypes in the new set $\tilde{G}\left(t\right)$ is defined by:


$$\begin{array}{l} \tilde{F}\left(t\right)=\tilde{F^{*}}\left(t\right)\cup \left\{{\tilde{f}}_{{ɤ}_{v}}\;:\;v\in \overset{\sim }{V}\left(t\right)\right\}\\ \;=\left\{{\tilde{f}}_{1}\left(t\right),...,{\tilde{f}}_{m\left(t\right)+q\left(t\right)}\left(t\right)\right\} \end{array}$$

where $\tilde{F^{*}}$ is the set of frequencies F^*^ updated by deducing the frequency of genomes that were mutated and $\left\{{\tilde{f}}_{m\left(t\right)+1},\ldots ,{\tilde{f}}_{m\left(t\right)+q\left(t\right)}\left(t\right)\right\}$ is the vector of the *q*(*t*) genotype frequencies; ∀*k* = *m*(*t*) + 1, ..., *m*(*t*) + *q*(*t*), *f*_*k*_(*t*) = 1.

Then, genotypes whose frequencies are zero in $\tilde{G}\left(t\right)$ are deleted, identical genotypes are aggregated and their frequencies are summed. Thus, we obtain the set *G*(*t*) of genotypes present in the host at time *t*, after the growth and mutation stages, and *F*(*t*) the frequency vector of these genotypes.

#### 2.2.3. Shuffling process

Here, we describe how we build probabilities P^*^(*t* − 1) equal to standardized noisy versions of the proportions *P*(*t* − 1), introduced in Equation (3). Beyond the effect of mutation, genotype frequencies may vary due to other mechanisms such as natural selection and random genetic drift (Lande, 1976). To implicitly account for the effect of such mechanisms into our within-host pathogen evolutionary model, we incorporate a shuffling process into the model. This process consists of drawing genotype proportions with an over-dispersion to eventually simulate the extra multiplication of low-proportion genotypes and/or the reduced multiplication of high-proportion genotypes.

Let *P* denote a vector of proportions that sum to one [typically, *P*(*t* − 1) in Section 2.2.1]. The vector of proportions P^*^ provided by the shuffling process applied to *P* is obtained by noising *P* with a centered Gaussian distribution:


(4) $$\begin{array}{l} \tilde{P}|P\sim N\;(P,\sigma^{2}) \end{array}$$

where $\sigma^{2}=\gamma_{1}\times P^{\gamma_{2}}\times {\left(1-P\right)}^{\gamma_{3}}$ (γ_1_, γ_2_, γ_3_ ≥ 0); cutting $\tilde{P}$ off: $\hat{P}=\min\left\{1,\max\left(0,\tilde{P}\right)\right\}$; and re-scaling $\hat{P}$:


(5) $$\begin{array}{l} P^{*}=\frac{1}{\sum_{i=1}^{n}{\hat{p}}_{i}}\hat{P} \end{array}$$

where $\hat{P}=\left({\hat{p}}_{1},\ldots ,{\hat{p}}_{n}\right)$, *n* ∈ ℕ^*^. The effects of the shuffling parameters (γ_1_, γ_2_, γ_3_) are detailed in Supplementary Text 1. Briefly, the larger γ_1_, the larger the noise; the smaller γ_2_, the more some low-proportion genotypes may reach high frequencies; the smaller γ_3_, the more some high-proportion genotypes may reach low frequencies.

#### 2.2.4. Elimination of lethal genomes

The proportion of lethal mutations incurred by a viral genome lies between 0.2 and 0.4 for viruses infecting hosts from different kingdoms (animal, plant, bacteria; Sanjuán, 2010). For vesicular stomatitis virus (VSV), an animal virus for which this value is known, the proportion of lethal mutations is 0.4 (Sanjuán et al., 2004). Hence, we account for a reference proportion α = 0.4 of lethal mutations by discarding the genomes with mutations in the first 40% of the nucleotide positions along the sequence; the other mutations are considered neutral. To allow the assessment of the presence or absence of lethal-genome elimination given the viral kinetics, the proportion, and the frequency of each genotype are re-scaled after the lethal-genome elimination step, such that the sum of proportions is one and the sum of frequencies equals *V*(*t*).

### 2.3. Genetic diversity indices

To measure the level of genetic diversity of the pathogen population within an infected host at each generation *t*, we used several diversity indices. The first three indices are haplotype diversity indices that depend on genotype abundance (Morris et al., 2014). The fourth index quantifies pairwise genetic distances that depend on sequence variation.

#### 2.3.1. Richness (*R*)

The richness estimator *R*(*t*) is the simple count of different genotypes existing at time *t*. It is equal to *n*(*t*). This index is therefore highly sensitive to rare genotypes.

#### 2.3.2. Shannon index (*H*′)

The Shannon diversity index is calculated as follows:


(6) $$\begin{array}{l} H^{\prime }\left(t\right)=-\overset{R\left(t\right)}{\underset{i=1}{\sum }}p_{i}\left(t\right)\text{ log}\left(p_{i}\left(t\right)\right) \end{array}$$

where *R*(*t*) is the number of existing genotypes (richness) at time *t* and *p*_*i*_(*t*) is the proportion of the *i*-th genotype at time *t*. This index is both sensitive to rare and abundant genotypes.

#### 2.3.3. Gini-Simpson index (*D*)

The Gini-Simpson index also depends on the genotype proportions and is defined as follows:


(7) $$\begin{array}{l} D\left(t\right)=1-\overset{R\left(t\right)}{\underset{i=1}{\sum }}p_{i}^{2}\left(t\right) \end{array}$$

where *R*(*t*) and *p*_*i*_(*t*) are defined as for Equation (6). This index is sensitive to abundant genotypes.

#### 2.3.4. Jukes-Cantor distance (*JC*)

Pairwise indices are grounded on the comparison of the sequences of each pair of sequences. Here, we used the Jukes-Cantor distance (Jukes and Cantor, 1969) to evaluate the within-host genetic diversity. Supposing that the rate of nucleotide substitution is the same between any pair of nucleotides, the Jukes-Cantor distance is defined in the following way:


(8) $$\begin{array}{l} \bar{d}\left(t\right)={𝔼}_{ij}\left[d\left(g_{i}\left(t\right),g_{j}\left(t\right)\right)\right] \end{array}$$

where *i* and *j* represent two genotypes drawn randomly, independently, and uniformly from the genotype space and *d*(*g*_*i*_(*t*), *g*_*j*_(*t*)) is given by:


$$\begin{array}{l} d\left(g_{i}\left(t\right),g_{j}\left(t\right)\right)=-\frac{3}{4}\text{log}\left(1-\frac{4}{3}p\left(g_{i}\left(t\right),g_{j}\left(t\right)\right)\right) \end{array}$$

with *p*(*g*_*i*_(*t*), *g*_*j*_(*t*)) the mean pairwise distance (p-distance) between the two sequences *g*_*i*_(*t*) and *g*_*j*_(*t*). This p-distance is the proportion of nucleotide sites at which *g*_*i*_(*t*) and *g*_*j*_(*t*) differ, and it is estimated by ${\hat{p}}_{i,j}\left(t\right)=n_{i,j}\left(t\right)/L$, *n*_*i, j*_(*t*) being the number of nucleotide differences between *g*_*i*_(*t*) and *g*_*j*_(*t*).

### 2.4. Simulation setting

In order to study the impact of the demo-genetic factors on the within-host genetic diversity, we measured the genetic diversity of pathogen populations by the above-mentioned indices during 10 generations. Each pathogen population is characterized by a set of viral genotypes generated *via* our evolutionary model where the length of each genetic sequence was set to *L* = 330 nucleotides and the mutation rate was set to μ = 10^−5^ mutation per nucleotide per generation, as default values. These populations differ in the demo-genetic characteristics that are included through the kinetic model, the shuffling process and the elimination of lethal genomes. We remind that kinetic parameters are specified in Tables 1, 2. Default values of genetic parameters are specified in Table 3. This table also indicates how default values are varied for each figure displaying diversity dynamics. For each demo-genetic scenario, we performed 100 independent simulations of the temporal dynamics of the within-host population.

**Table 3:** List of parameters included in the genetic component of the model and their default values (top part of the table); values actually taken by these parameters, set of kinetic parameters and number of generations per day (G/d) for each figure displaying diversity dynamics (bottom part of the table).

**Symbol**	**Definition**						**Default value**	
*L*	Length of genome fragment						330	
μ	Mutation rate per nucleotide per generation						10^−5^	
γ_1_	Scale parameter in the noise variance of the shuffling process						0.8	
γ_2_	First shape parameter in the noise variance of the shuffling process						0.4	
γ_3_	Second shape parameter in the noise variance of the shuffling process						70	
α	Proportion of lethal mutations						0.4	
**Figure**	**L**	**μ**	**γ_1_**	**γ_2_**	**γ_3_**	**α**	**Kinetic parameters**	**G/d**
Figure 3	330	10^−5^	{0.8, NA}	{0.4, NA}	{70, NA}	{0, 0.4}	Tables 1, 2	1
Supplementary Figure 1	330	10^−5^	NA	NA	NA	(0.2, 0.4)	Tables 1, 2	1
Supplementary Figure 2	330	10^−5^	0.8	0.4	70	(0.2, 0.4)		1
Figure 4	330	10^−5^	0.8	(0, 1)	70	0	Tables 1, 2	1
Supplementary Figure 3	330	10^−5^	0.8	(0,1)	70	0.4	Tables 1, 2	1
Supplementary Figure 4	330	10^−5^	(0,1)	0.4	70	0	Tables 1, 2	1
Supplementary Figure 5	330	10^−5^	0.8	0.4	(0,100)	0	Tables 1, 2	1
Figure 5	330	(5 × 10^−7^, 5 × 10^−5^)	0.8	0.4	70	0.4	Tables 1, 2	1
Figure 6	(30, 1,200)	10^−5^	0.8	0.4	70	0.4	Tables 1, 2	1
Supplementary Figure 6	330	(5 × 10^−7^, 5 × 10^−5^)	0.8	0.4	70	0	Tables 1, 2	1
Supplementary Figure 7	(30, 1,200)	10^−5^	0.8	0.4	70	0	Tables 1, 2	1
Supplementary Figure 8	330	(5 × 10^−7^, 5 × 10^−5^)	NA	NA	NA	0.4	Tables 1, 2	1
Supplementary Figure 9	330	10^−5^	{0.8, NA}	{0.4, NA}	{70, NA}	{0, 0.4}	Tables 1, 2	1
Supplementary Figure 10	330	10^−5^	0.8	0.4	70	0.4	Tables 1, 2 + scaling to vary *V*_max_	1
Supplementary Figure 11	330	10^−6^	{0.8, NA}	{0.4, NA}	{70, NA}	{0, 0.4}	Table 1 for shared parameters	1
Supplementary Figure 12	330	10^−5^	0.8	0.4	70	0.4	Tables 1, 2	{1, 2}

For Supplementary Figure 11, kinetic parameters that are not shared (i.e., those specific to models with persistent infection and immune response) are given in Supplementary Figure 11 caption, and μ was set at 10^−6^ to keep the computation time reasonable for the model with persistent infection whose maximum viral load was larger than 5 × 10^6^. Unique value when the parameter is fixed; values within brackets when two values are used; values within parentheses when a range of values is used, the two values given providing the minimum and the maximum of the range.

### 2.5. Backbone of the algorithmic description of the model

Here, the model is summarized *via* an algorithmic description. Details and justifications are provided in the previous subsections.

1. Set parameter values;
2. Set initial states (at time, or generation, *t* = 0) of the number of virions *V*(0), the family of genotypes *G*(0) and the genotype frequencies *F*(0);
3. Compute the number of virions *V*(*t*) from *t* = 1 to *t* = 10 with the kinetic model;
4. For time *t* from 1 to 10,
  *Growth of genotypes conditional on the kinetics*
  (a) Compute previous proportions of genotypes *P*(*t* − 1) = *V*(*t* − 1)^−1^*F*(*t* − 1);
  (b) If the shuffling process is applied, add noise to *P*(*t* − 1) with a centered Gaussian distribution: $\tilde{P}\left(t-1\right)|P\left(t-1\right)\sim N\left\{P\left(t-1\right),\sigma^{2}\right\}$, cut $\tilde{P}\left(t-1\right)$ off: $\hat{P}\left(t-1\right)=\min1,\max\left\{0,\tilde{P}\left(t-1\right)\right\}$; and rescale $\hat{P}\left(t-1\right)$: $P^{*}\left(t-1\right)={\left(\sum_{i=1}^{n}{\hat{p}}_{i}\left(t-1\right)\right)}^{-1}\hat{P}\left(t-1\right)$;
    Else keep *P* unchanged: P^*^(*t* − 1) = *P*(*t* − 1);
  (c) Draw the frequency vector of the *n*(*t* − 1) genotypes constituting the *G*(*t* − 1) family after the growth stage: *F*′(*t*)∣P^*^(*t* − 1), *V*(*t*) ~ Multinomial [*V*(*t*), P^*^(*t* − 1)];
  (d) Remove genotypes with zero-frequencies and update genotype frequencies accordingly; updated genotypes and genotype frequencies are denoted G^*^(*t*) and F^*^(*t*);
  *Mutations of genomes*
  (e) For genome, or virion, *v* from 1 to *V*(*t*), draw the number of mutations *N*_*v*_(*t*) ~ Binomial(*L*, μ) and let $V\left(t\right)=\left\{v=1,\ldots ,V\left(t\right):N_{v}\left(t\right)>0\right\}$ denote the set of genomes undergoing at least one mutation;
  (f) For genome v∈V(t), draw *N*_*v*_(*t*) indices from {1, …, *L*} and update the nucleotide corresponding to each index by drawing a new nucleotide from the set {A,C,G,T} excluding the current value of the nucleotide;
  (g) If the elimination of lethal genomes is applied, discard the genomes with mutations in the first 40% of the nucleotide positions along the sequence and update genotype frequencies accordingly;
  (h) Remove genotypes whose frequencies are zero, aggregate identical genotypes and update genotype frequencies accordingly; updated genotypes and their frequencies are denoted *G*(*t*) and *F*(*t*), respectively;
5. For time *t* from 0 to 10, compute the four diversity indices based on variables derived from {*G*(*t*), *F*(*t*)}.

## 3. Results

Table 3 provides the model parametrizations that were used for all the sets of simulations corresponding to all the figures mentioned in the results section, the parameters that vary in each figure, and the ranges of variation of these parameters.

### 3.1. Cross-effects of the viral kinetic, the shuffling process, and the elimination of lethal genomes

**Figure 3:**
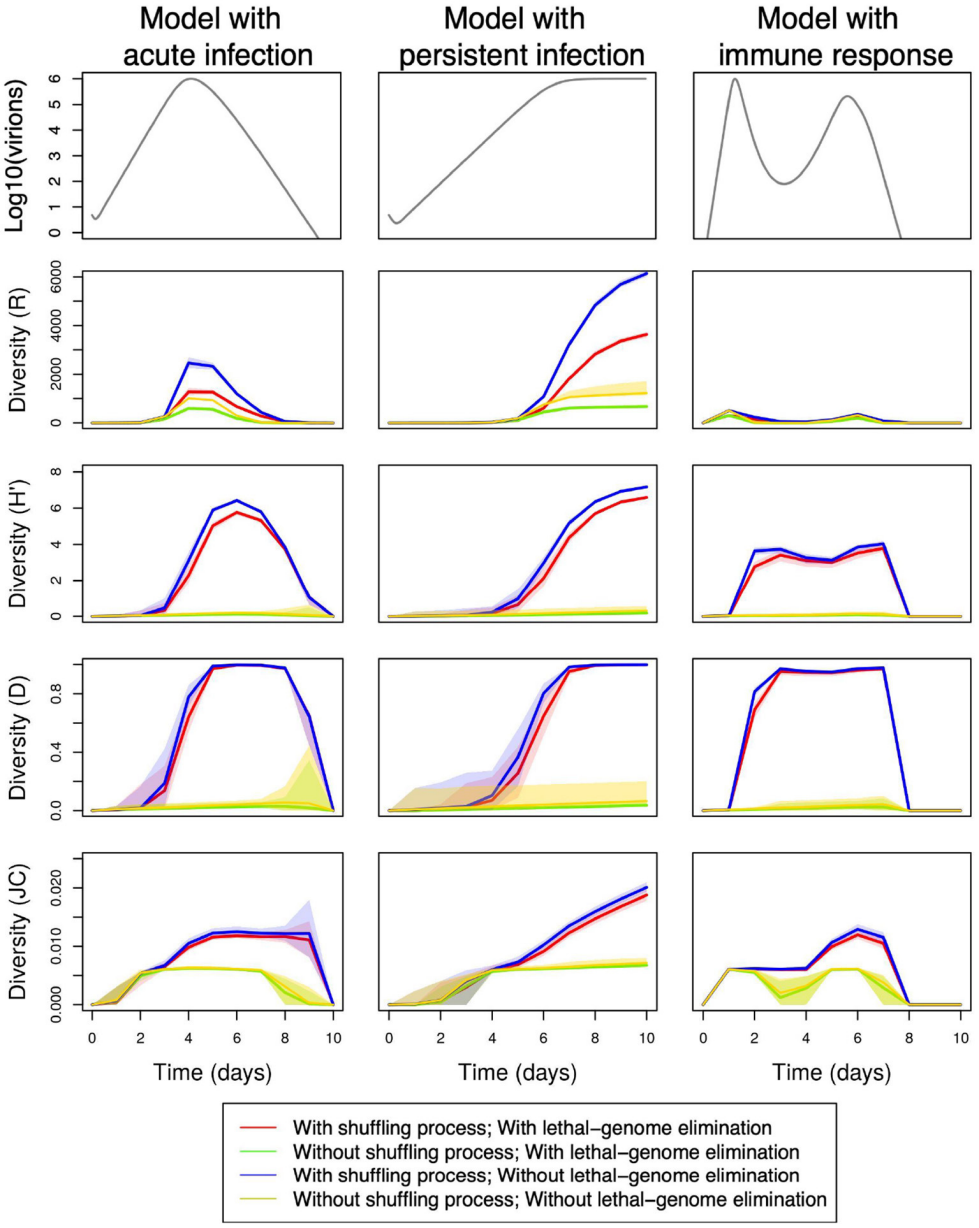
Temporal variations in within-host genetic diversity under various demo-genetic conditions, namely with or without the shuffling process [(γ_1_, γ_2_, γ_3_) = (0.8, 0.4, 70) or (NA, NA, NA)] and with or without the elimination of lethal genomes (α = 0.4 or 0); See Table 3 for full parameter specification. Row 1: variation in within-host virion quantity under the models with acute infection (column 1), persistent infection (column 2), and immune response (column 3). Rows 2–5: variation in within-host genetic diversity measured by richness (*R*), Shannon (*H*′), Gini-Simpson (*D*), and Jukes-Cantor (*JC*) indices, respectively. Shaded areas delimit the 95% pointwise confidence envelopes of the diversity curves under all demo-genetic conditions.

Figure 3 shows, for three different viral kinetics, the temporal evolution of the genetic diversity of the viral population within a host during an infection, computed from 100 replicates for each kinetic and each model configuration (i.e., with/without shuffling process; with/without lethal genome elimination). The diversity is assessed with the four indices described in Section 2.3: richness (*R*), Shannon (*H*′), Gini-Simpson (*D*), and Jukes-Cantor (*JC*). The kinetic models, which quantify the temporal variation of the viral load during the infection, are those presented in Section 2.1: the acute model, the persistent infection model and the model with an immune response. The simulations are performed with default parameter values, namely the kinetic parameters given in Tables 1, 2, α = 0.4 when lethal genomes are eliminated and (γ_1_, γ_2_, γ_3_) = (0.8, 0.4, 70) when the shuffling process is applied.

The four diversity indices are more or less smoothed and delayed versions of the temporal dynamics of virions. We however note that the number of different genotypes is strongly reduced by a fast onset of the immune response (index *R*, column 3).

Figure 3 shows that promoting non-equilibrium and fast variations with the shuffling process induces a marked increase in the within-host genetic diversity, whatever the index, even with lethal genomes (red and blue lines). In addition, the presence of the shuffling process results in major qualitative changes in the within-host diversity measured by the *H*′ and *D* indices, and to a lesser extent by the *JC* index. This statement can be observed in more details by comparing Supplementary Figures 1, 2, which show the temporal changes in the four diversity indices when the proportion of lethal mutations α varies between 0.2 and 0.4, either in the absence of the shuffling process (Supplementary Text 1) or in its presence (Supplementary Text 2).

Figure 3 also shows, as intuitively expected, that negative selection against lethal mutations (red and green lines) reduces the richness (*R*) by 60% both in the presence and in the absence of the shuffling process (i.e., when viral multiplication probabilities are noised). In contrast, lethal genome elimination seems to have little impact on Shannon (*H*′), Gini-Simpson (*D*), and Jukes-Cantor (*JC*) diversity indices. Supplementary Figures 1, 2 essentially confirms this observation.

### 3.2. Fast changes in genotype proportions

**Figure 4:**
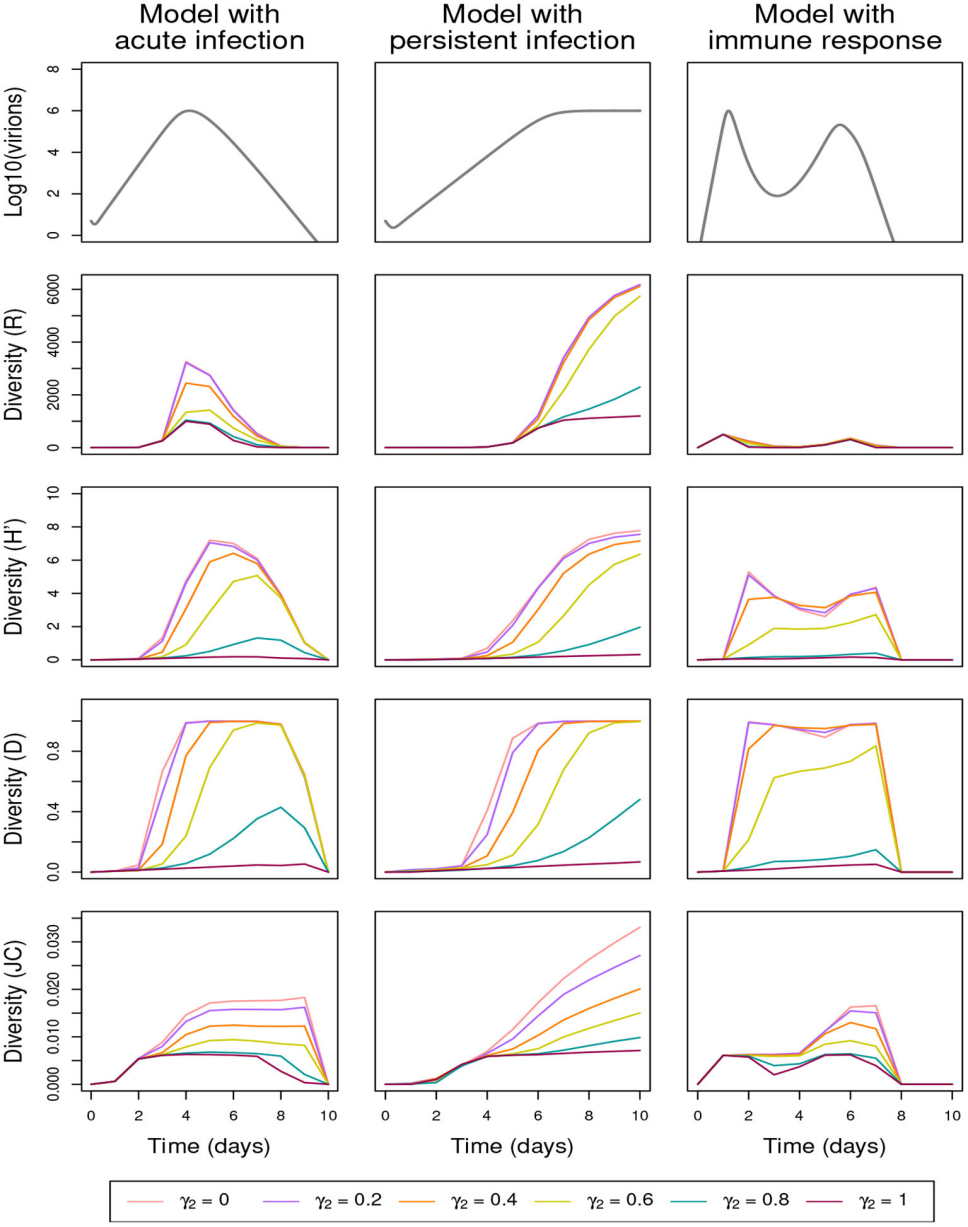
Effect of the shuffling parameter γ_2_ on within-host genetic diversity in the absence of lethal-genome elimination (α = 0), the other shuffling parameters being equal to their default values (γ_1_, γ_3_) = (0.8, 70); See Table 3 for full parameter specification. Row 1: variation in within-host virion quantity under the models with acute infection (column 1), persistent infection (column 2), and immune response (column 3). Rows 2–5: variation in within-host genetic diversity measured by richness (*R*), Shannon (*H*′), Gini-Simpson (*D*), and Jukes-Cantor (*JC*) indices, respectively.

In the shuffling process, the enhancement of low-proportion genotypes is governed in particular by parameter γ_2_: the lower γ_2_, the larger the dispersion of the noise affecting genotype proportions in the multiplication stage and, consequently, the faster some low-proportion genotypes may reach large proportions. Figure 4 and Supplementary Figure 3 (both displaying the effect of the variation of γ_2_ but corresponding respectively to simulations without and with lethal genome elimination) show that variation in γ_2_ generates significantly different temporal profiles for all the diversity indices. The overdispersion obtained with small γ_2_ increases the number of genotypes (*R*), the probability of substitutions (*JC*), and the evenness in genotypes abundance (*H*′). In addition, small γ_2_ values rapidly lead to a maximum Gini-Simpson diversity (*D*). The two other shuffling parameters, γ_1_ and γ_3_, have much less influence (apart for γ_1_ = 0) on the diversity indices (see Supplementary Figures 4, 5, which display the effect of the variation of γ_1_ and γ_3_, respectively, both in the absence of lethal-genome elimination).

### 3.3. Changes in the number of mutations

**Figure 5:**
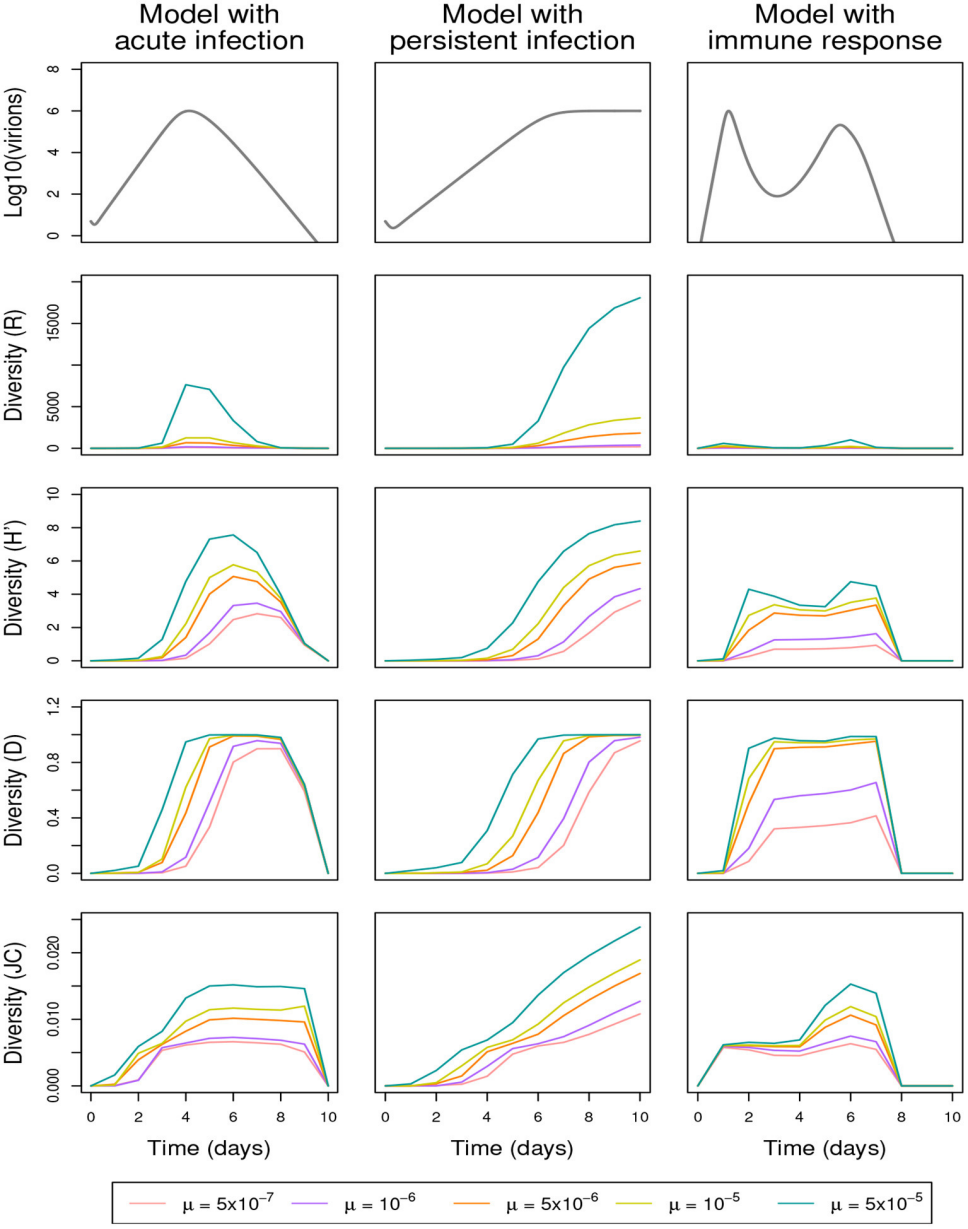
Effect of the mutation rate μ on within-host genetic diversity in the presence of the shuffling process [(γ_1_, γ_2_, γ_3_) = (0.8, 0.4, 70)] and the elimination of lethal genomes (α = 0.4); See Table 3 for full parameter specification. Row 1: variation in within-host virion quantity under the models with acute infection (column 1), persistent infection (column 2), and immune response (column 3). Rows 2–5: variation in within-host genetic diversity measured by richness (*R*), Shannon (*H*′), Gini-Simpson (*D*) and Jukes-Cantor (*JC*) indices, respectively. See Table 3 for full parameter specification.

**Figure 6:**
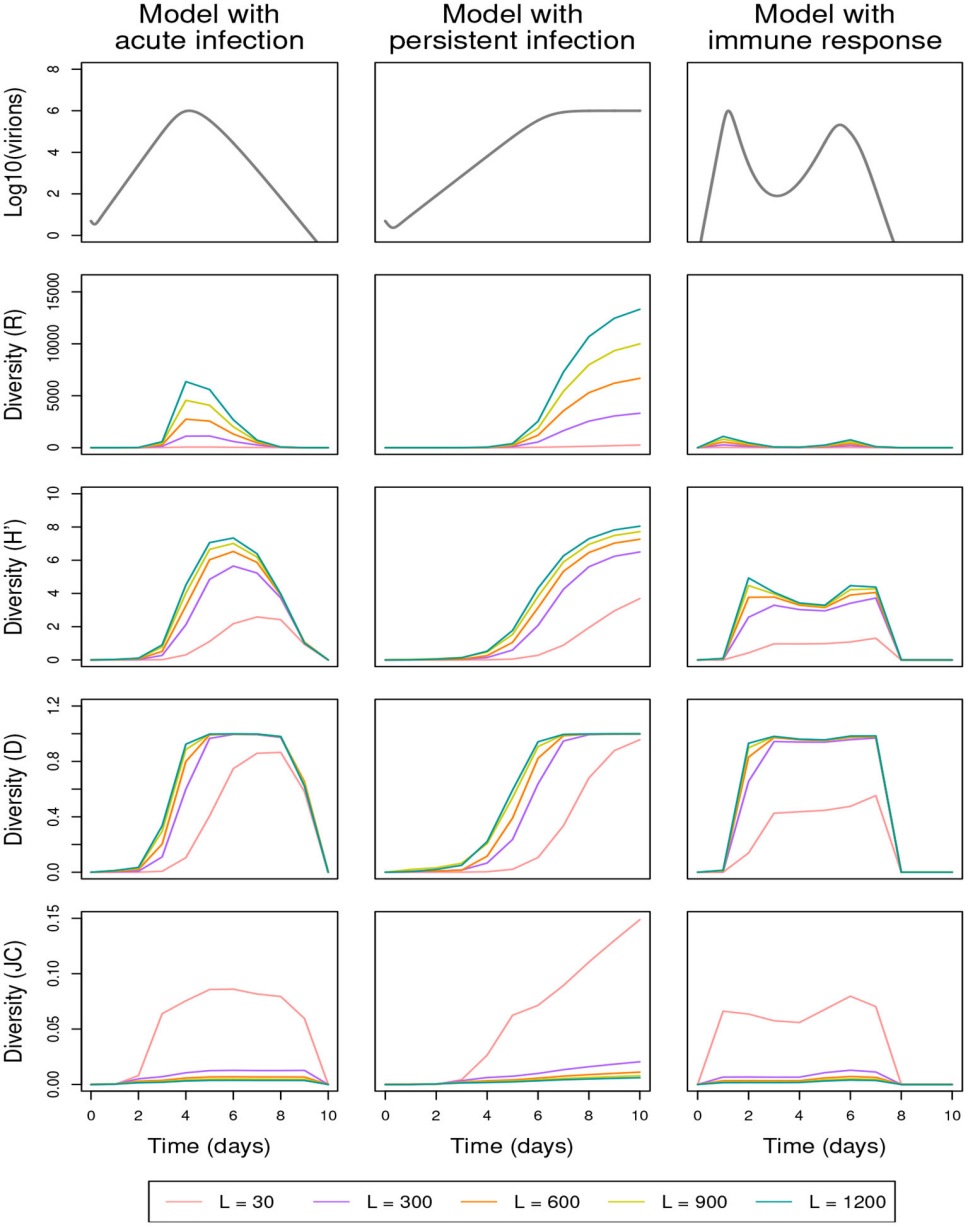
Effect of the genetic sequence size *L* on within-host genetic diversity in the presence of the shuffling process [(γ_1_, γ_2_, γ_3_) = (0.8, 0.4, 70)] and the elimination of lethal genomes (α = 0.4); See Table 3 for full parameter specification. Row 1: variation in within-host virion quantity under the models with acute infection (column 1), persistent infection (column 2), and immune response (column 3). Rows 2–5: variation in within-host genetic diversity measured by richness (*R*), Shannon (*H*′), Gini-Simpson (*D*), and Jukes-Cantor (*JC*) indices, respectively. See Table 3 for full parameter specification.

The proportion of mutated genomes increases with the mutation rate μ and the genome size *L*. By applying the shuffling process and eliminating the lethal genomes or not, Figures 5, 6 and Supplementary Figures 6–8 show that the three diversity indices *R*, *H*′, and *D* are affected in a qualitatively similar manner by increasing μ or *L* (Figures 5, 6, respectively display the effect of the variation of μ and *L*, in the presence of the shuffling process and the elimination of lethal genomes; Supplementary Figures 6, 7 are analogous to Figures 5, 6, but do not include lethal-genome elimination; Supplementary Figure 8 is analogous to Figure 5, but does not include the shuffling process). These effects are similar to the one obtained by decreasing γ_2_ (Figure 4). To refine this observation, the increased diversity of genotypes obtained for higher values of μ or *L* is reflected by an increased richness (*R*) and a faster increase of the Gini-Simpson diversity (*D*) up to its maximum. Even for high values of mutation rate and genome size, the immune response mitigates the replication of new genotypes, leading to a very low richness (*R*) and a low evenness (*H*′).

As expected, the proportion of nucleotide differences (*JC*) increases with the mutation rate μ (Figure 5), while it decreases when the sequence size *L* increases (Figure 6), even in the absence of removal of lethal genomes (Supplementary Figure 7). The *JC* index is computed among unique sequences which, for a given mutation rate, are less numerous when sequences are shorter. Thus, the *JC* curves corresponding to *L* = 30 reach significantly higher values than the curves obtained for longer sequences, and the following curves gradually and slightly lower from *L* = 300 to *L* = 1, 200 nucleotides.

## 4. Discussion

### 4.1. Discussion of the results

In this work, we introduced a stochastic model to simulate within-host pathogen evolution during an infection in order to outline the demographic and genetic factors shaping viral within-host genetic diversity. Our explicit model developed in a forward framework allows us to monitor temporal changes (i.e., across viral generations) in within-host genetic diversity computed under various demo-genetic scenarios. This model is able to generate very diverse within-host scenarios in terms of viral load and genetic diversity as illustrated in Section 3. Demographic effects are considered mainly through the kinetic model quantifying the temporal variation of the viral load. Genetic effects are considered through mutation and replication processes approximately mimicking natural selection and genetic drift. These processes are based, in particular, on the elimination of lethal genomes (leading to negative selection) and the shuffling of genotype proportions generating over-dispersion with respect to multinomial draws (corresponding to genetic drift and positive selection). We observed a major impact of the shuffling process on within-host genetic diversity, both qualitatively and quantitatively, whatever the diversity index. There are two explanations to this observation: firstly, the shuffling process favors the number of genotypes (i.e., the richness *R*) despite the mass at zero of the noisy proportions (see Section 2.2.3); secondly, the shuffling process favors the presence of a larger number of abundant genotypes, as particularly illustrated with Shannon (*H*′) and Gini-Simpson (*D*) indices that are sensitive to abundant genotypes. Thus, by coupling the model that we propose with a host-to-host transmission model, we could obtain a flexible basis to challenge, in very diverse settings, the methods that reconstruct transmission trees between hosts on the basis of multiple virus sequences collected from each host (e.g., De Maio et al., 2018; Alamil et al., 2019). The host-to-host transmission model may specifically include an impact of viral load in the source host at transmission time (i) on transmission probability and/or (ii) on the initial viral load (and hence, on the subsequent dynamics) in the recipient host.

In contrast to the Wright-Fisher model considering that the total pathogen population size is constant (Fisher, 1923; Wright, 1931; Imhof and Nowak, 2006) and to the Worby and Read model (Worby and Read, 2015) assuming that the size of the pathogen population converges to an attraction function *via* the sum of binomial jumps, in our approach, virion-quantity changes during an infection are explicitly modeled (and hence controlled), and we can use many existing viral kinetic models found in the specialized literature. Predictions from the neutral theory, which highlight the importance of population size and genetic drift, provide a useful null model allowing to assess the occurrence and strength of selection on intra-host genetic diversity in rapidly evolving pathogens (Nelson and Hughes, 2015; Frost et al., 2018; Lauring, 2020). However, the succession of demographic processes (e.g., founder effect, expansion, bottleneck) and their consequences on genetic diversity during the course of an infection can be quite complex. Thus, by accounting for temporal variation in virus load (under different kinetic assumptions) and contrasting diversity indices, we can investigate the relative importance of viral load and genetic drift in shaping intra-host genetic diversity dynamics. With this in mind, we specifically observed a non-monotonic relationship between pathogen population size and genetic diversity. In Figure 3, this non-monotonic relationship is mostly exemplified by the comparison between the richness index (*R*) at the peak population size across the three kinetic models, or by the contrasting patterns of the different diversity indices for the model with immune response (and to a lesser extent of the *JC* index for the persistent infection model). Even for the acute infection model where all diversity curves seem to grossly correlate with viral load, there are delays between the peak population size and the peaks in diversity indices, and these delays often induce huge differences in diversity indices for similar viral loads, as further illustrated by Supplementary Figure 9). This may result from the complex interplay between diversity accumulation through time and changes in the size of the pathogen population. Analyzing and confronting the variations of different diversity indices in further analyses may provide some clues on the major processes shaping genetic diversity across time through main and interaction effects.

Interactions between genetic and demographic forces have been pointed out in numerous studies: pathogen population size can impact mutational robustness (Elena et al., 2007) as well as robustness to random genetic drift (Kuo et al., 2009; Didelot et al., 2016; LaBar and Adami, 2017) and selection intensity (Gutiérrez et al., 2012; Didelot et al., 2016; Frickel et al., 2018), which directly affects the composition of the viral population. Our study also illustrates such interactions (the word “interaction” being considered in its statistical meaning, i.e., the effect of a factor on a dependent variable, here a diversity index, changes according to the values of one or more other factors). Consider as an example the demographic force consisting of the immune response included in the kinetic model with immune response. The level of within-host genetic diversity and the mutation rate are known to be positively correlated (Xu et al., 2019; Castellano et al., 2020) and we clearly see this with the assessment of richness and Jukes-Cantor indices in Figure 5. However, the immune response reduces, in general, the impact of mutation on diversity and reduces, in particular, the evenness of mutant genotypes (Shannon index). By considering that the immune response *de facto* induces an additional selective pressure, the negative effect of the immune response on diversity can be viewed as a manifestation of the overall quick response of rapidly mutating viruses (such as RNA viruses) to selection (Domingo and Holland, 1997; Holmes, 2009; Sanjuán, 2010). From a methodological point of view, a global sensitivity analysis and accompanying graphs (Saltelli et al., 2008; Wainwright et al., 2014) may be applied in a further study to deepen the multidimensional understanding of the covariation between parameters and diversity indices.

### 4.2. Perspectives in modeling

In the analysis presented in this paper, we compared diversity for three fixed viral kinetics that were standardized by setting the same value for the maximum number of virions reached over the 10-day study period (similarly, one may constrain the cumulative number of virions over 10 days to be the same). This choice allowed us to mitigate the effect of population size on diversity (which is major, as illustrated by Supplementary Figure 10) and, hence, to investigate the effect of the shape of the viral kinetics on diversity. However, beyond this objective, we aim in further studies to use our model to numerically test methods for reconstructing transmissions between hosts. In this perspective, one may simulate scenarios where the virus has intrinsic growth characteristics that are modulated from one host to another by different immunity components (Blanco-Rodriguez et al., 2021) and/or different values for parameters driving immunity strength (Hernandez-Vargas and Velasco-Hernandez, 2020). Supplementary Figure 11 somehow illustrates such a configuration at the within-host level: using the three models of Section 2.1 and setting the same values for the shared parameters, the original model components induce differences in the viral kinetics, especially in the maximum viral load, and subsequently on diversity dynamics. This is obviously a schematic setting, and host-to-host variations in the shared parameters may be included to match previous work (e.g., Baccam et al., 2006; Pawelek et al., 2012) where parameters (even those not directly related to immunity) are separately estimated for different hosts. In addition, other kinetic models (with or without shared parameters) adapted to the disease and the population under study may deserve to be considered, as discussed below.

Our model can easily incorporate more advanced kinetic models of the number of virions and, hence, be used, e.g., to study within-host pathogen diversity in the presence of alternative immunity processes (Blanco-Rodriguez et al., 2021), antiviral treatment (Beauchemin et al., 2008; Smith and Perelson, 2011), chronic infection (Perelson and Nelson, 1999; Pinky and Dobrovolny, 2017), co-infection (Pinky and Dobrovolny, 2017, 2020), multiple target cells (Wang et al., 2020), variation of virion infectivity over time (Smith and Ribeiro, 2010; Vaidya et al., 2010; Beauchemin and Handel, 2011), co-receptor switch (Alizon and Boldin, 2010) and virion loss due to cell entry (Beauchemin et al., 2008; Gonçalves et al., 2020). We implemented a few of these models in the R code MoWPP accompanying this manuscript in addition to the three kinetic models presented in Section 2.1. Another perspective is to provide alternative choices in the genetic component of our hybrid model. The basic substitution model used here may notably be replaced by more realistic mutation processes (Kimura, 1980; Tavaré, 1986; Nishimaki and Sato, 2019), which would constrain the frequencies of different sequence modifications and might possibly modify the observed diversity patterns. While viral load was modeled as a continuous process (using ODEs) like most of the standard virus kinetics models found in the literature, sampling of virus genomes during replication and within-host infection was modeled as a discrete process for numerical tractability, with genotype growth and genome mutation occurring at a discrete time step (once per day). Modifying this time step while keeping the same virus kinetics impacts the resulting diversity dynamics, as illustrated by Supplementary Figure 12 where genotype growth and genome mutation happen twice a day, another biologically plausible generation time for viruses (Roizman, 1996). In this case, the level of genetic diversity increases or reaches its largest value more rapidly, because the processes that generate and increase diversity occur twice more often. Other perspectives would consist in including relative fitness depending on the genetic sequence or frequency-dependent selection (Sanjuán et al., 2004; Alizon and Boldin, 2010); note that our model, where genetics is modeled conditionally on demographics, effectively copes with variations in the relative fitness of variants, but would need to be adapted to handle variations in absolute fitness that can impact population size (i.e., the numbers of virions).

Beyond considering the way the model components are defined, models can be improved by using realistic parameter values and implementing goodness-of-fit procedures of the fitted model(s) to validate their components. The statistical estimation of the model parameters from host-level kinetic data and within-host genetic data is likely to be a challenge that first requires to identify the appropriate inference approach and level of data accuracy using models such as the one presented here. This point is further discussed in Supplementary Text 2.

## Data availability statement

No original raw data were generated for this study. The publicly available datasets analyzed in this study can be found at: https://doi.org/10.5281/zenodo.6783246.

## Author contributions

MA, GT, KB, and SS conceived the methodology and MA implemented it. MA, GT, KB, and SS analyzed model output, contributed to interpretation of results, and gave final approval for publication. MA prepared the initial draft of the manuscript. All authors participated to the writting of the subsequent versions. All authors contributed to the article and approved the submitted version.

## Funding

This work was funded by an ANR grant (SMITID project; ANR-16-CE35-0006).

## Conflict of interest

The authors declare that the research was conducted in the absence of any commercial or financial relationships that could be construed as a potential conflict of interest.

## Publisher's note

All claims expressed in this article are solely those of the authors and do not necessarily represent those of their affiliated organizations, or those of the publisher, the editors and the reviewers. Any product that may be evaluated in this article, or claim that may be made by its manufacturer, is not guaranteed or endorsed by the publisher.
