# Supplementary material for: Characterizing viral within-host diversity in fast and non-equilibrium demo-genetic dynamics
Source: Front Microbiol. 2022 Oct 5;13:983938. doi: 10.3389/fmicb.2022.983938 (PMC9581327; doi:10.3389/fmicb.2022.983938)
Supplement: Supplementary file 1 [file Presentation_1.pdf]

Characterizing viral within-host diversity in fast and non-equilibrium demo-genetic dynamics

—

Supporting information

Alamil M.[1,2,*], Thébaud G.[3], Berthier K.[4], and Soubeyrand S.[1,**]

[1]INRAE, BioSP, 84914 Avignon, France
[2]Present affiliation: Department of Mathematics and Computer Science, Alfaisal University, Riyadh, Kingdom of Saudi Arabia
[3]PHIM Plant Health Institute, INRAE, Univ Montpellier, CIRAD, Institut Agro, IRD, Montpellier, France
[4]INRAE, Pathologie Végétale, 84140, Montfavet, France
[*]Corresponding author: maryam.alamil@hotmail.com – malamil@alfaisal.edu
[**]Secondary corresponding author: samuel.soubeyrand@inrae.fr

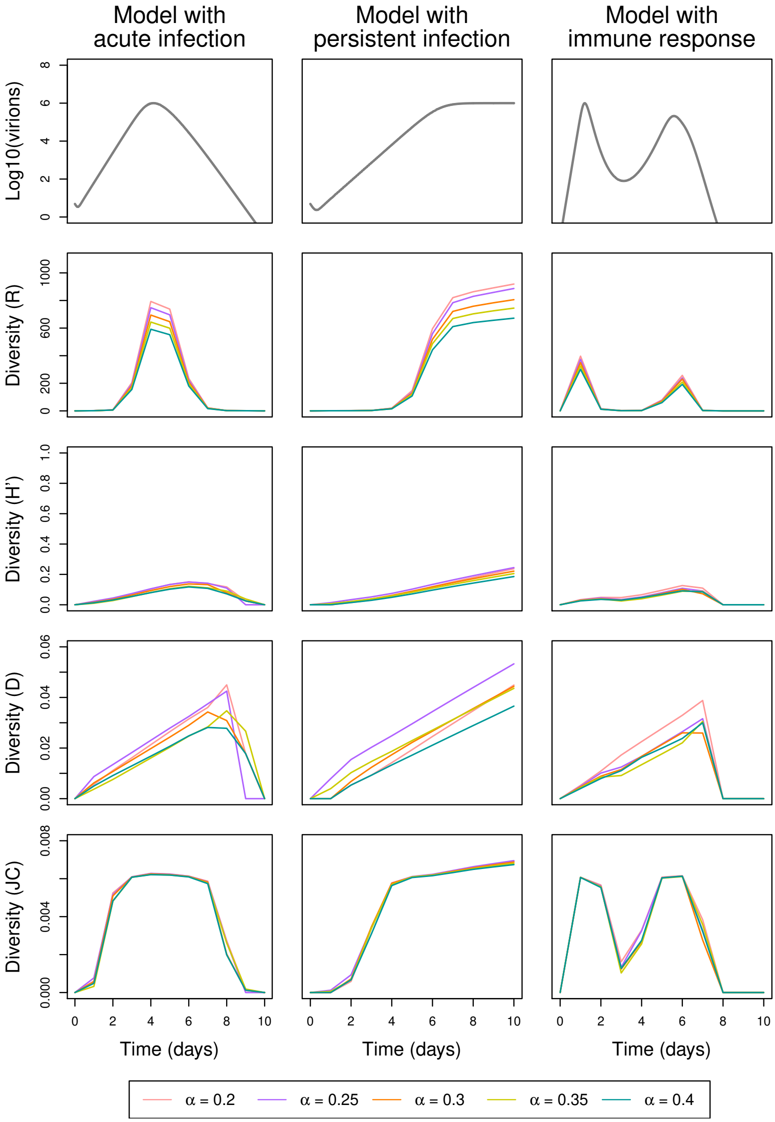

Figure S1: Effect of the proportion α of lethal mutations on within-host genetic diversity in the absence of the shuffling process; See table 3, main text, for full parameter specification. Row 1: variation in within-host virion quantity under the models with acute infection (column 1), persistent infection (column 2) and immune response (column 3). Rows 2–5: variation in within-host genetic diversity measured by richness (R), Shannon (H'), Gini-Simpson (D) and Jukes-Cantor (JC) indices, respectively.

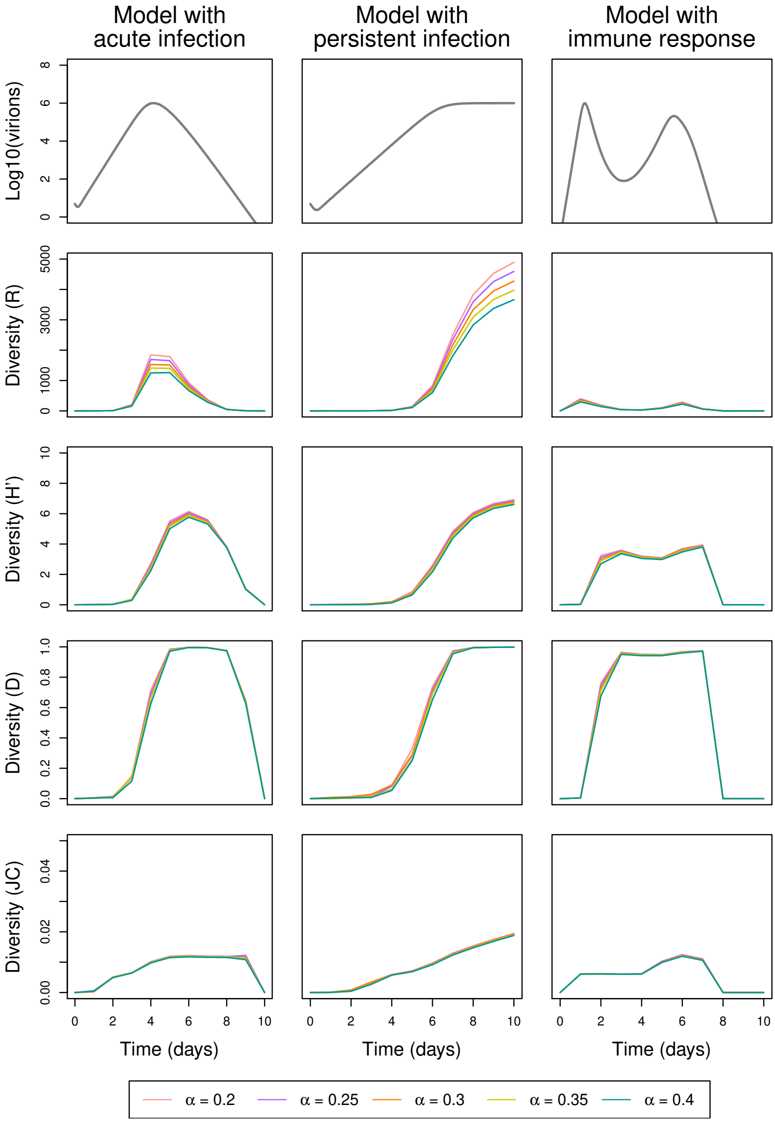

Figure S2: Effect of the proportion α of lethal mutations on within-host genetic diversity in the presence of the shuffling process $((\gamma_1, \gamma_2, \gamma_3) = (0.8, 0.4, 70))$; See table 3, main text, for full parameter specification. Row 1: variation in within-host virion quantity under the models with acute infection (column 1), persistent infection (column 2) and immune response (column 3). Rows 2–5: variation in within-host genetic diversity measured by richness (R), Shannon (H'), Gini-Simpson (D) and Jukes-Cantor (JC) indices, respectively.

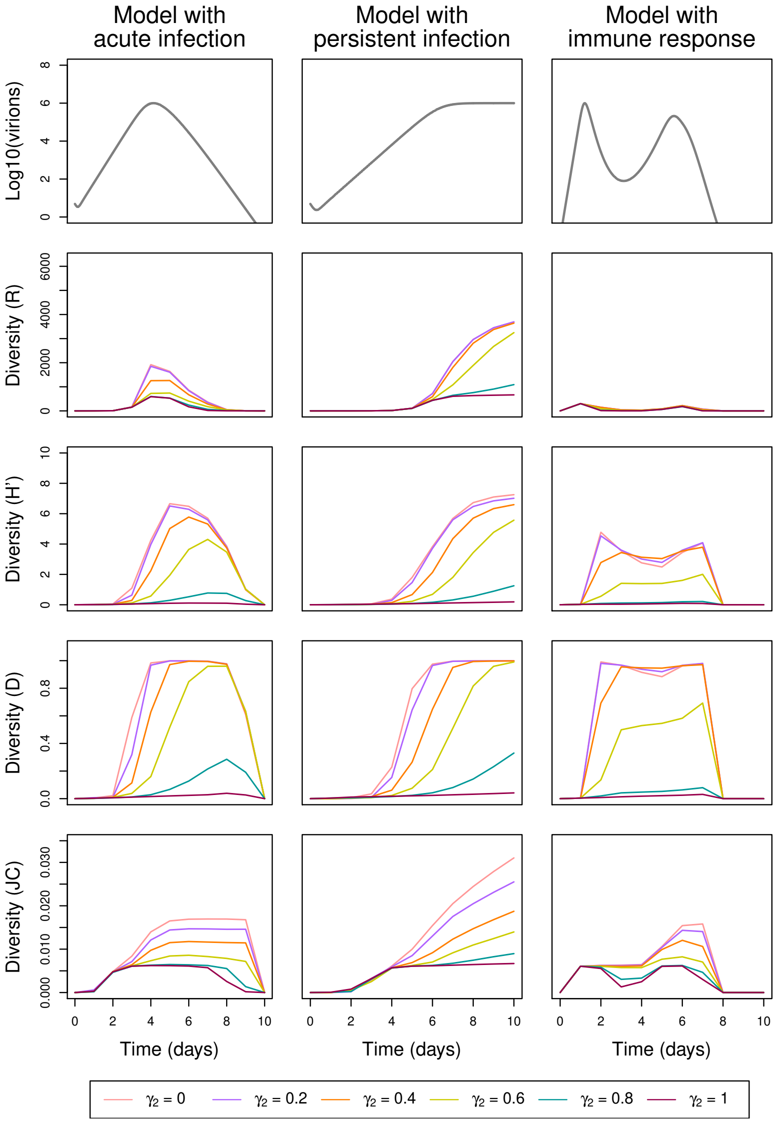

Figure S3: Effect of the shuffling parameter γ_2 on within-host genetic diversity in the presence of lethal-genome elimination ($\alpha = 0.4$), the other shuffling parameters being equal to their default values $(\gamma_1, \gamma_3) = (0.8, 70)$; See table 3, main text, for full parameter specification. Row 1: variation in within-host virion quantity under the models with acute infection (column 1), persistent infection (column 2) and immune response (column 3). Rows 2–5: variation in within-host genetic diversity measured by richness (R), Shannon (H'), Gini-Simpson (D) and Jukes-Cantor (JC) indices, respectively.

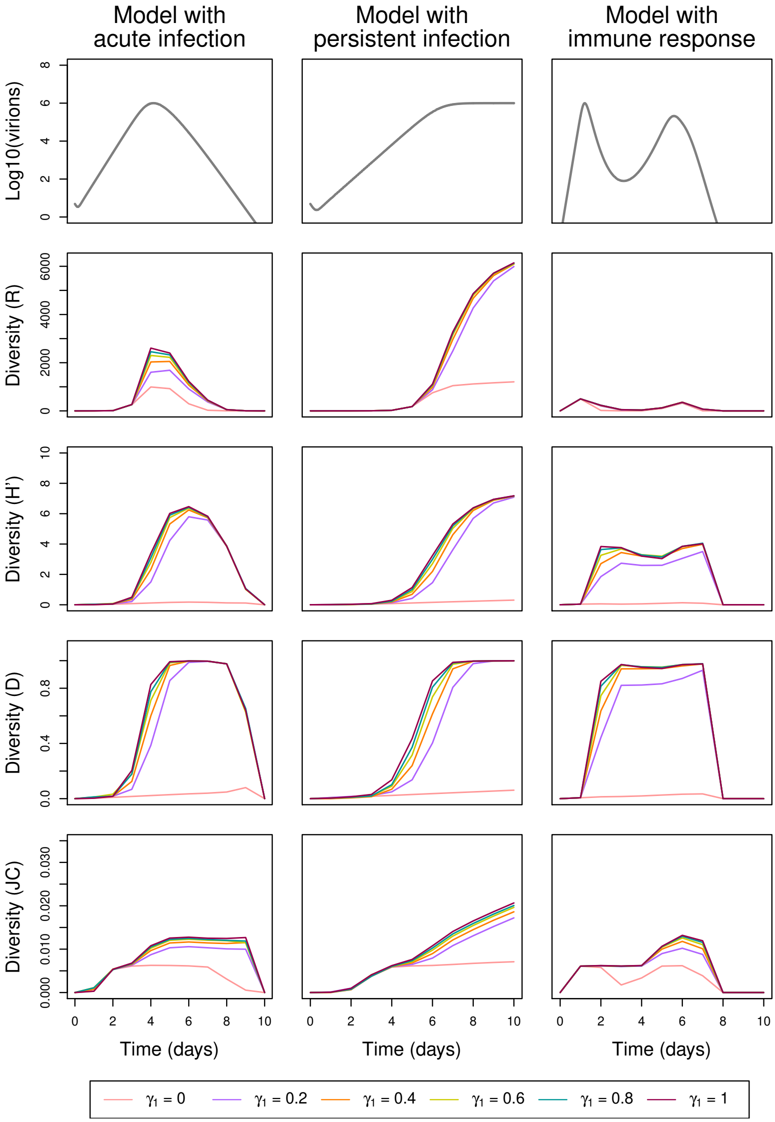

Figure S4: Effect of the shuffling parameter γ_1 on within-host genetic diversity in the absence of lethal-genome elimination ($\alpha = 0$), the other shuffling parameters being equal to their default values $(\gamma_2, \gamma_3) = (0.4, 70)$; See table 3, main text, for full parameter specification. Row 1: variation in within-host virion quantity under the models with acute infection (column 1), persistent infection (column 2) and immune response (column 3). Rows 2–5: variation in within-host genetic diversity measured by richness (R), Shannon (H'), Gini-Simpson (D) and Jukes-Cantor (JC) indices, respectively.

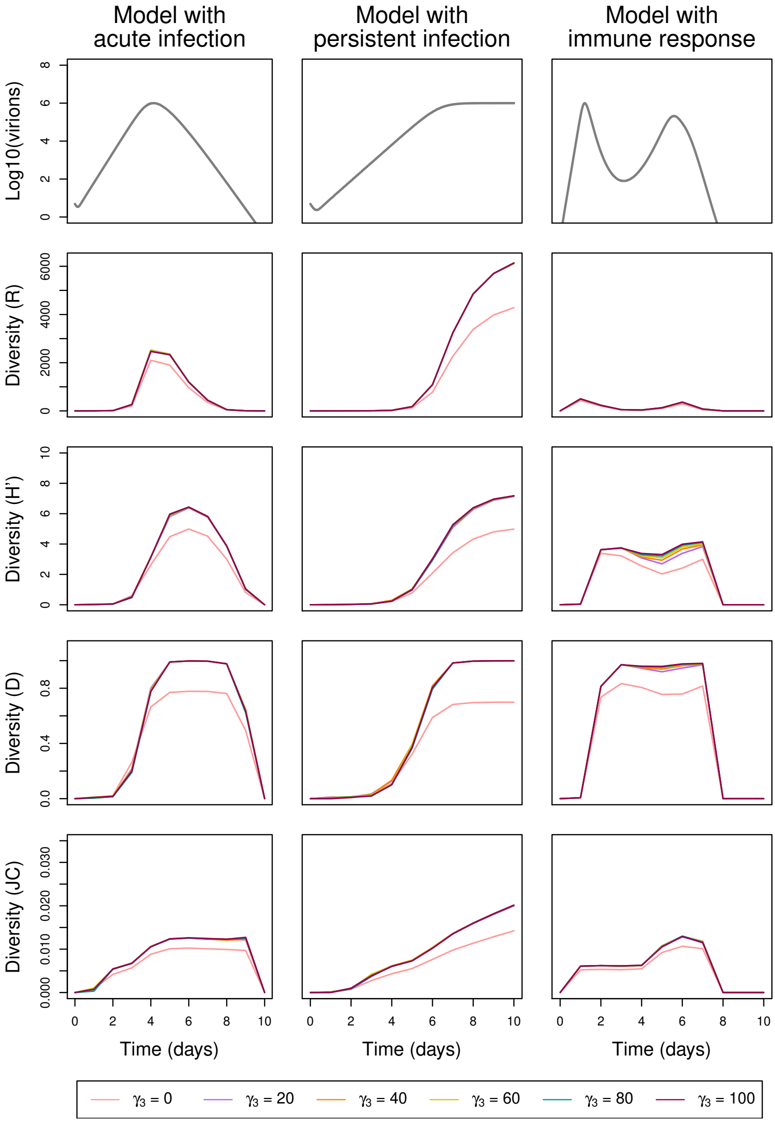

Figure S5: Effect of the shuffling parameter γ_3 on within-host genetic diversity in the absence of lethal-genome elimination ($\alpha = 0$), the other shuffling parameters being equal to their default values $(\gamma_1, \gamma_2) = (0.8, 0.4)$; See table 3, main text, for full parameter specification. Row 1: variation in within-host virion quantity under the models with acute infection (column 1), persistent infection (column 2) and immune response (column 3). Rows 2–5: variation in within-host genetic diversity measured by richness (R), Shannon (H'), Gini-Simpson (D) and Jukes-Cantor (JC) indices, respectively.

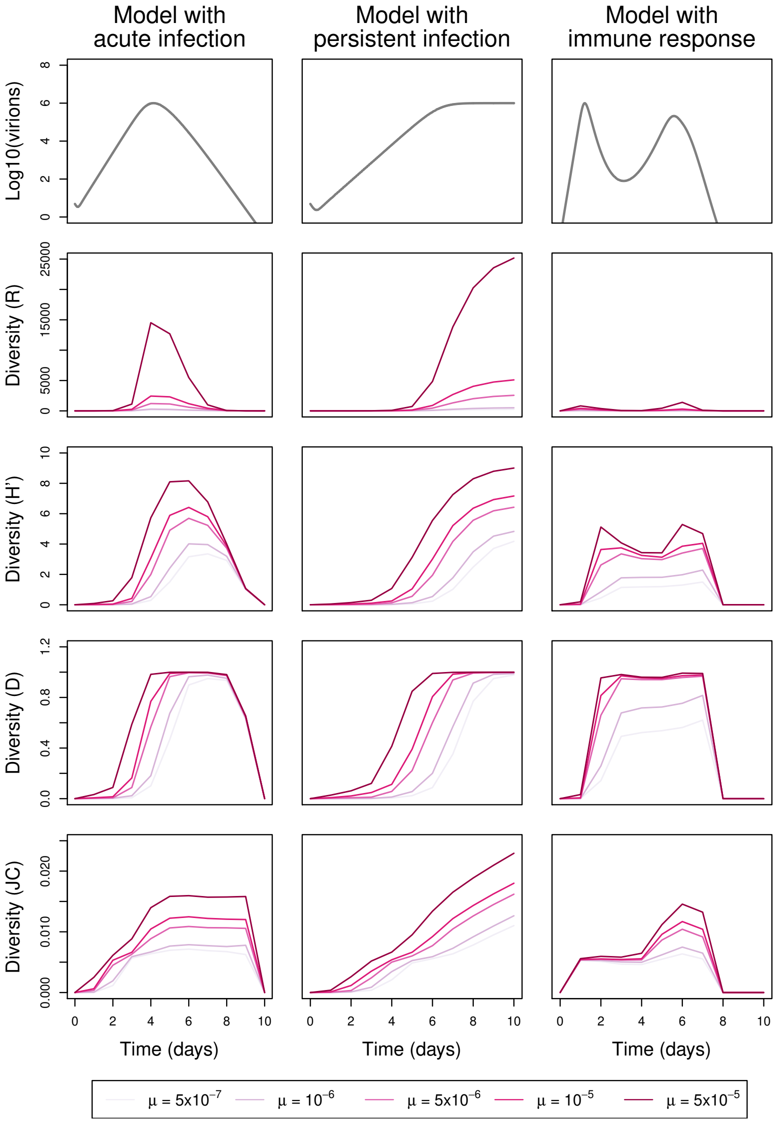

Figure S6: Effect of the mutation rate μ on within-host genetic diversity in the presence of the shuffling process $((\gamma_1, \gamma_2, \gamma_3) = (0.8, 0.4, 70))$ but without the elimination of lethal genomes $(\alpha = 0)$; See table 3, main text, for full parameter specification. Row 1: variation in within-host virion quantity under the models with acute infection (column 1), persistent infection (column 2) and immune response (column 3). Rows 2–5: variation in within-host genetic diversity measured by richness (R), Shannon (H'), Gini-Simpson (D) and Jukes-Cantor (JC) indices, respectively.

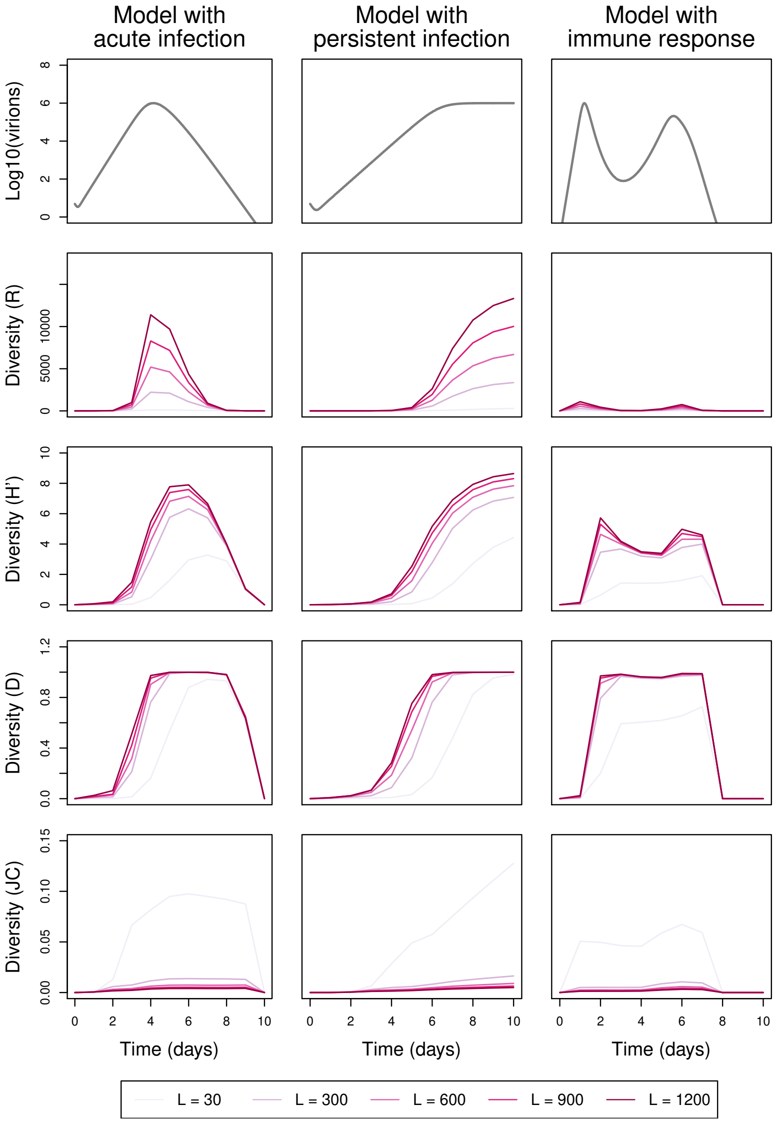

Figure S7: Effect of the genetic sequence size L on within-host genetic diversity in the presence of the shuffling process $((\gamma_1, \gamma_2, \gamma_3) = (0.8, 0.4, 70))$ but without the elimination of lethal genomes ($\alpha = 0$); See table 3, main text, for full parameter specification. Row 1: variation in within-host virion quantity under the models with acute infection (column 1), persistent infection (column 2) and immune response (column 3). Rows 2–5: variation in within-host genetic diversity measured by richness (R), Shannon (H'), Gini-Simpson (D) and Jukes-Cantor (JC) indices, respectively.

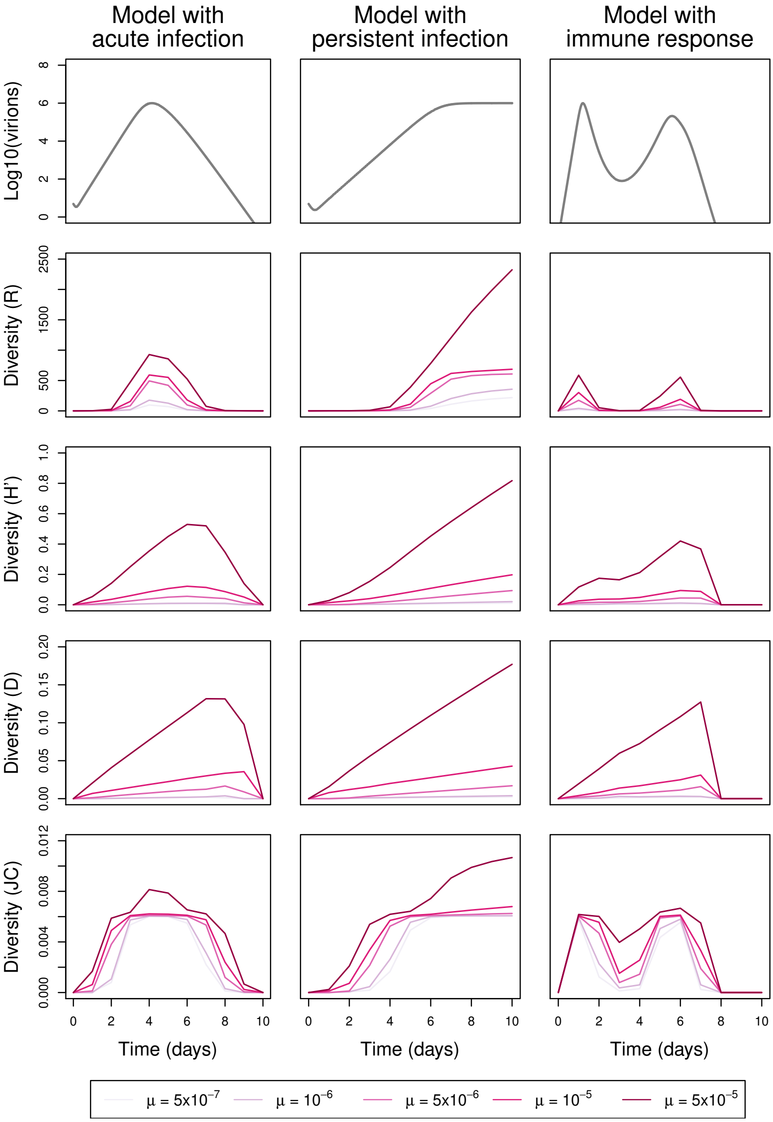

Figure S8: Effect of the mutation rate μ on within-host genetic diversity in the absence of the shuffling process but with the elimination of lethal genomes ($\alpha = 0.4$); See table 3, main text, for full parameter specification. Row 1: variation in within-host virion quantity under the models with acute infection (column 1), persistent infection (column 2) and immune response (column 3). Rows 2–5: variation in within-host genetic diversity measured by richness (R), Shannon (H'), Gini-Simpson (D) and Jukes-Cantor (JC) indices, respectively.

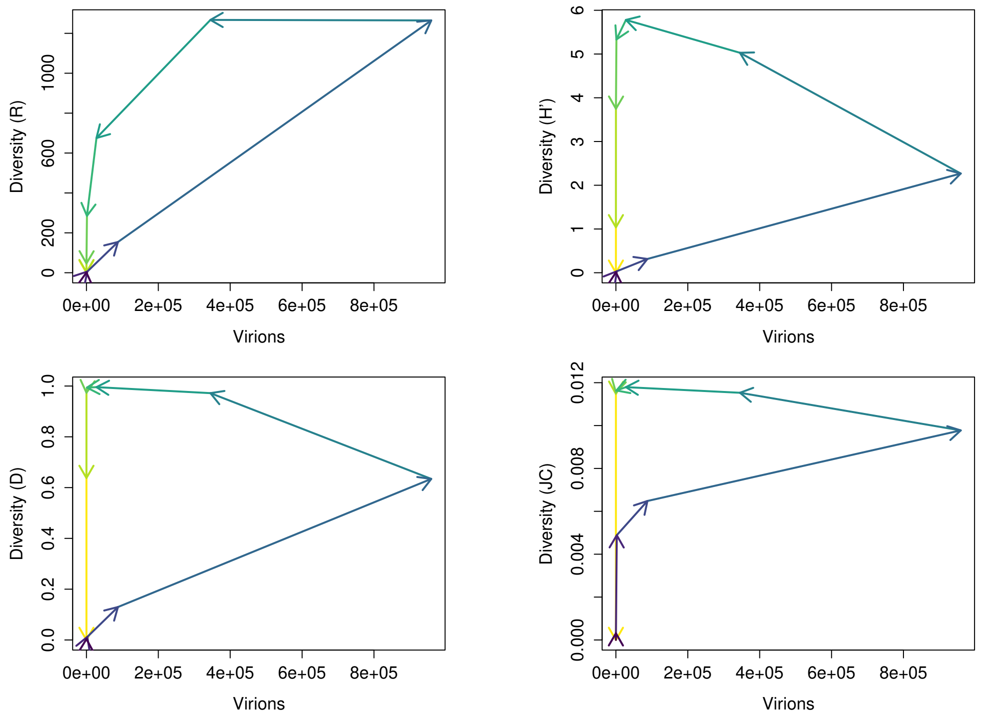

Figure S9: Illustration of the non-monotonic behavior of diversity indices with respect to viral load. The arrows and the color palette give temporal information of the evolution of the pair 'viral load–diversity index', from day 0 (dark blue) to day 10 (yellow). These curves correspond to viral load and diversity shown for the acute infection model with the shuffling process and the elimination of lethal genomes, figure 3, main text. If richness (R) is roughly monotonic with respect to viral load, this is not the case for Shannon (H'), Gini-Simpson (D) and Jukes-Cantor (JC) indices, which continue to increase for some time after the viral-load peak, and finally decrease when the pathogen population vanishes.

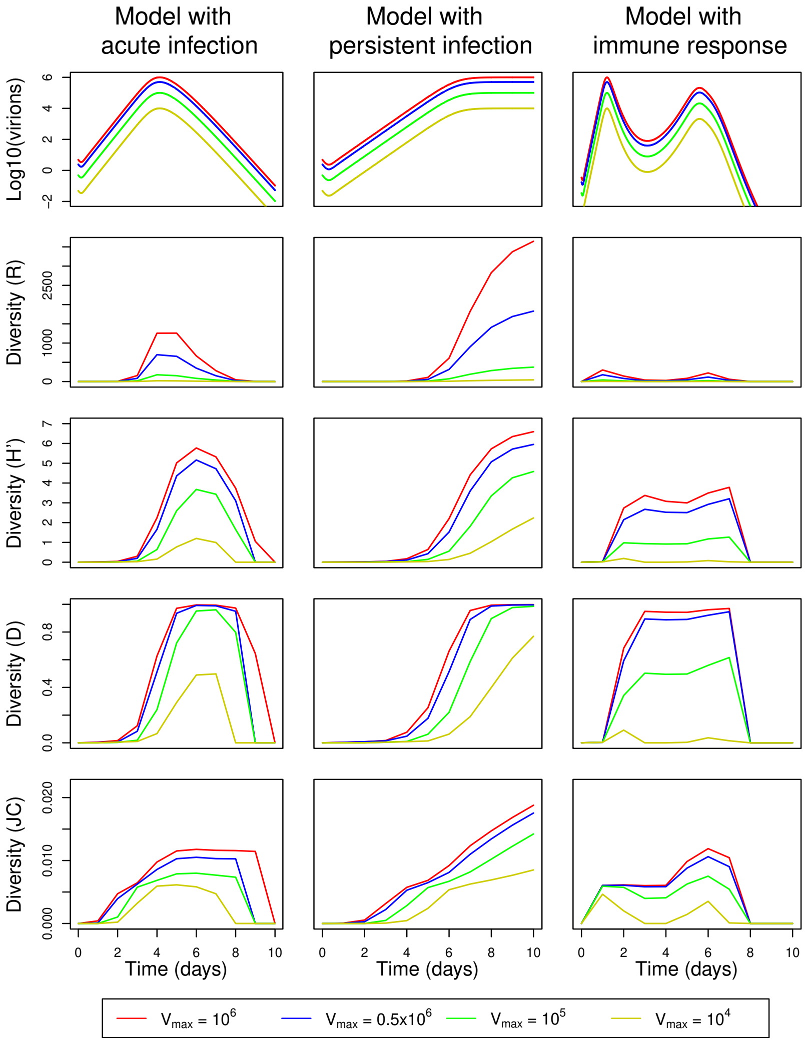

Figure S10: Effect of re-scaling the virion dynamics on within-host genetic diversity. Virion dynamics initially simulated with parameters provided by tables 1–2, main text, and then re-scaled by a multiplicative factor of 1, 0.5, 0.1 and 0.01 to obtain maximum viral load $V_{\max}$ equal to 10^6, 0.5×10^6, 10^5 and 10^4, respectively. Given these kinetics, simulations were performed with the shuffling process $((\gamma_1, \gamma_2, \gamma_3) = (0.8, 0.4, 70))$, the elimination of lethal genomes ($\alpha = 0.4$) and $(L, \mu) = (330, 10^{-5})$; see table 3 in main text for full parameter specification. Row 1: changes in within-host virion quantity predicted respectively by the three different kinetic models mentioned in Section 2.1. Rows 2-5: variation in within-host genetic diversity assessed by the four indices presented in Section 2.3, main text. Notes: Red curves in the diversity panels correspond to red curves in figure 3, main text. For the virion dynamics generated with the persistent infection model and multiplied by 0.01, the virion quantity at time 1 was too low for the genetic dynamics to be initialized and was therefore increased to 1 virion (the quantity of virions at time one was large enough in the other cases).

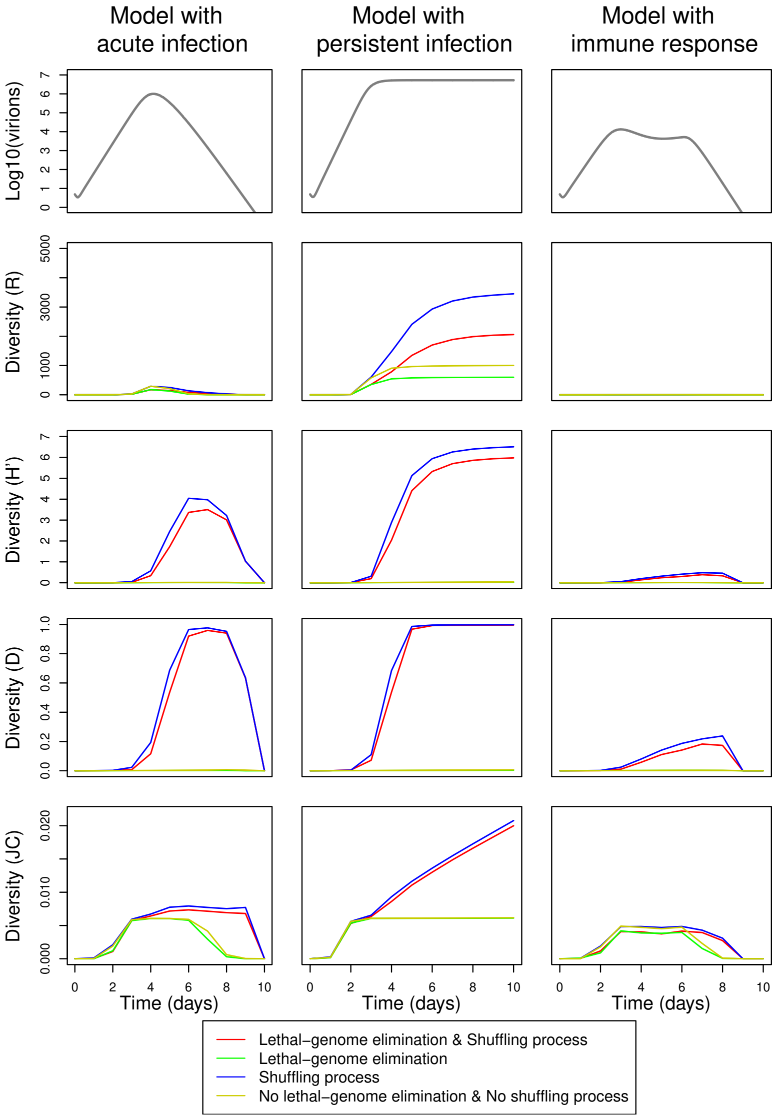

Figure S11: Temporal variations in within-host genetic diversity, as displayed by figure 3 (main text), when the virus has similar intrinsic growth characteristics in the three kinetic models. This condition was implemented as follows: for the three models, $(S(0), I_1(0), I_2(0), V(0), \beta, k, p, c,) = (4 \times 10^8, 0, 0, 4.9, 5.3 \times 10^{-6}, 4, 0.05, 3.8)$, as in table 1 (main text); for the acute infection, $\delta = 3.8$, as in table 1; for the persistent infection $\delta = 0$; for the immune response, $\delta_{\mathrm{I}} = 3.8$ (analogue of δ for the acute infection) and $(R(0), F(0), \phi, \rho, t_{\mathrm{I}}, \sigma_{\mathrm{I}}, m, q, d) = (0, 10, 10^{-4}, 1, 6, 4, 0.001, 0.01, 1)$ to obtain two peaks (however not very pronounced). With these sets of values, $V_{\max} = 10^6$ only for the acute infection, $V_{\max} \approx 5.3 \times 10^6$ for the persistent infection and $V_{\max} \approx 1.3 \times 10^4$ for the immune response model. Regarding the genetic component of the model, $(\mu, L) = (10^{-6}, 330)$, $\alpha = 0.4$ in case of lethal-genome elimination and $(\gamma_1, \gamma_2, \gamma_3) = (0.8, 0.4, 70)$ when the shuffling process was applied. See figure 3 for the description of the panels and curves.

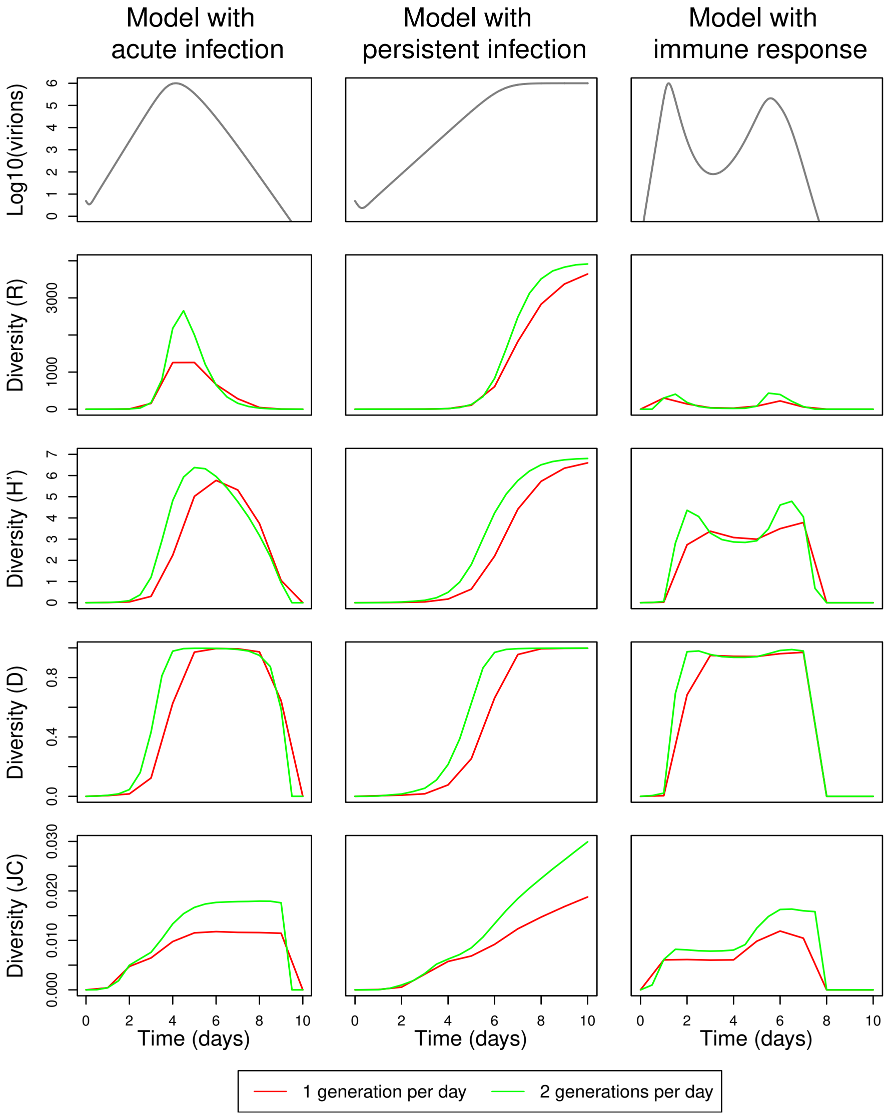

Figure S12: Effect of the generation time-step, at which genotype growth and genome mutation occur, on within-host genetic diversity. Simulations performed with either one or two generation(s) per day, with the shuffling process $((\gamma_1, \gamma_2, \gamma_3) = (0.8, 0.4, 70))$, with the elimination of lethal genomes $(\alpha = 0.4)$, $(L, \mu) = (330, 10^{-5})$ and kinetic parameters provided by tables 1–2, main text. Row 1: variation in within-host virion quantity under the models with acute infection (column 1), persistent infection (column 2) and immune response (column 3). Rows 2–5: variation in within-host genetic diversity measured by richness (R), Shannon (H'), Gini-Simpson (D) and Jukes-Cantor (JC) indices, respectively.

S1 Supporting text: Specific study of the shuffling process

Our model includes a component that accounts for episodes of natural selection and strong genetic drift by enhancing, e.g., the growth of low-frequency variants. This component, called shuffling process (Main text, Section 2.2.3), is constructed by noising the variant proportions P with the Gaussian distribution $\mathcal{N}(P, \gamma_1 \times P^{\gamma_2} \times (1-P)^{\gamma_3})$ of mean P and variance depending on P and three non-negative parameters γ_1, γ_2 and γ_3, by applying a two-side cut-off $\min\{1, \max\{0, \cdot\}\}$, and then by dividing the resulting variables by their sum to get a vector of probabilities. To gain insight into the impact of the shuffling parameters on the variant proportions, we apply the noising process to a set of 100 probabilities (summing to one) obtained by drawing a vector from the Dirichlet distribution with rate vector $\alpha = (\alpha_1, ..., \alpha_{100})$, using $\alpha_1 = ... = \alpha_{80} = 0.02$ and $\alpha_{81} = ... = \alpha_{100} = 0.06$.

Figure S13 displays a realization of the Dirichlet draw and illustrates how the proportions of the dominant variants may decrease and the weights of a few low-proportion variants may increase by noising the vector of probabilities with $(\gamma_1, \gamma_2, \gamma_3) = (0.8, 0.4, 70)$. Using the same values for $(\gamma_1, \gamma_2, \gamma_3)$, Figure S14 displays the evolution of the noise variance (namely $\gamma_1 \times P^{\gamma_2} \times (1-P)^{\gamma_3}$) with respect to the initial probability value P generated with the Dirichlet distribution. The highest variance corresponds to intermediate (but rather low) values of P. Note that this variance profile is prior to the re-scaling. To illustrate the variance obtained after the re-scaling, we applied the shuffling process 10000 times to the same vector of Dirichlet probabilities (by taking into account the two-side cut-off and the division by the sum) for assessing the distributions of the noisy versions of the maximum probability ($p_{\max}$; i.e. the maximum of the set of probabilities generated with the Dirichlet distribution), the minimum positive probability ($p_{\min}$; i.e. the minimum of the set of probabilities generated with the Dirichlet distribution) and the probability corresponding to the maximum variance ($p_{\text{var}_{\max}}$; i.e. the probability of the maximum variance value in Figure S14). These distributions are displayed in Figure S15 and show how variable the probabilities are in one step (i.e., one generation). For evaluating the potential evolution of probabilities throughout 10 generations, we sequentially repeated the shuffling process 10 times. In other words, starting with the initial set of probabilities generated with the Dirichlet distribution, at each generation we applied the shuffling process to the shuffled probabilities of the previous generation (by taking into account the two-side cut-off and the division by the sum). This process was repeated 10000 times. Figure S16 gives the evolution of the maximum probability, the minimum positive probability and the probability that has undergone the greatest variation. We notice a decrease of the maximum probability

value. In contrast, we observe an increase in average of the minimum probability and the maximum-variation probability.

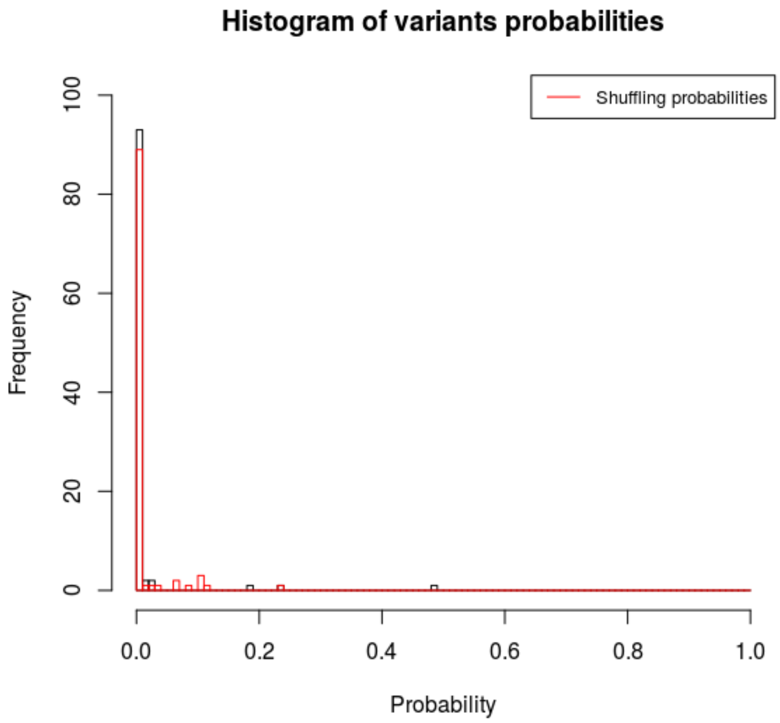

Figure S13: Distributions of initial (black) and shuffled (red) probabilities.

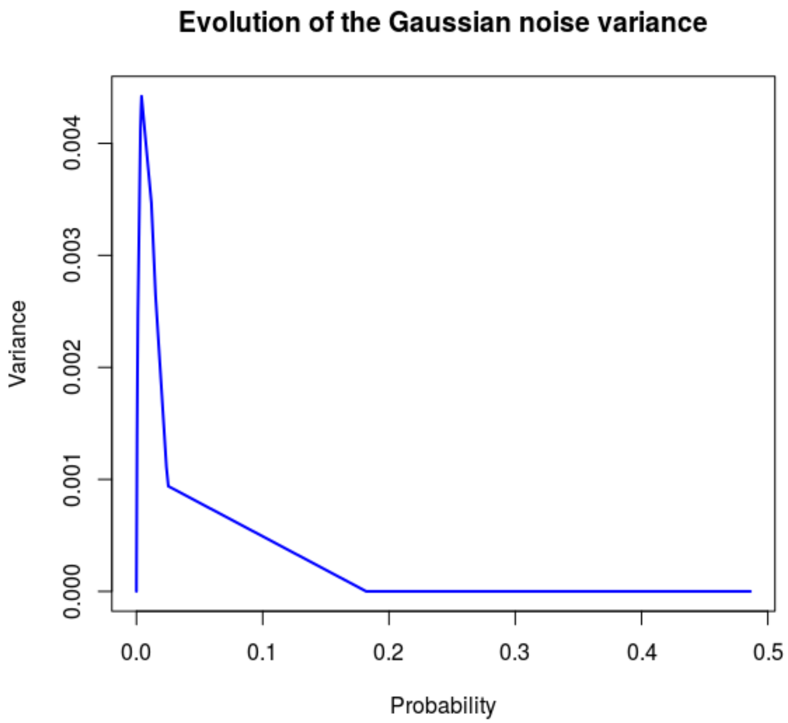

Figure S14: Values of the Gaussian noise variances with respect to the initial probability values sampled from the Dirichlet distribution.

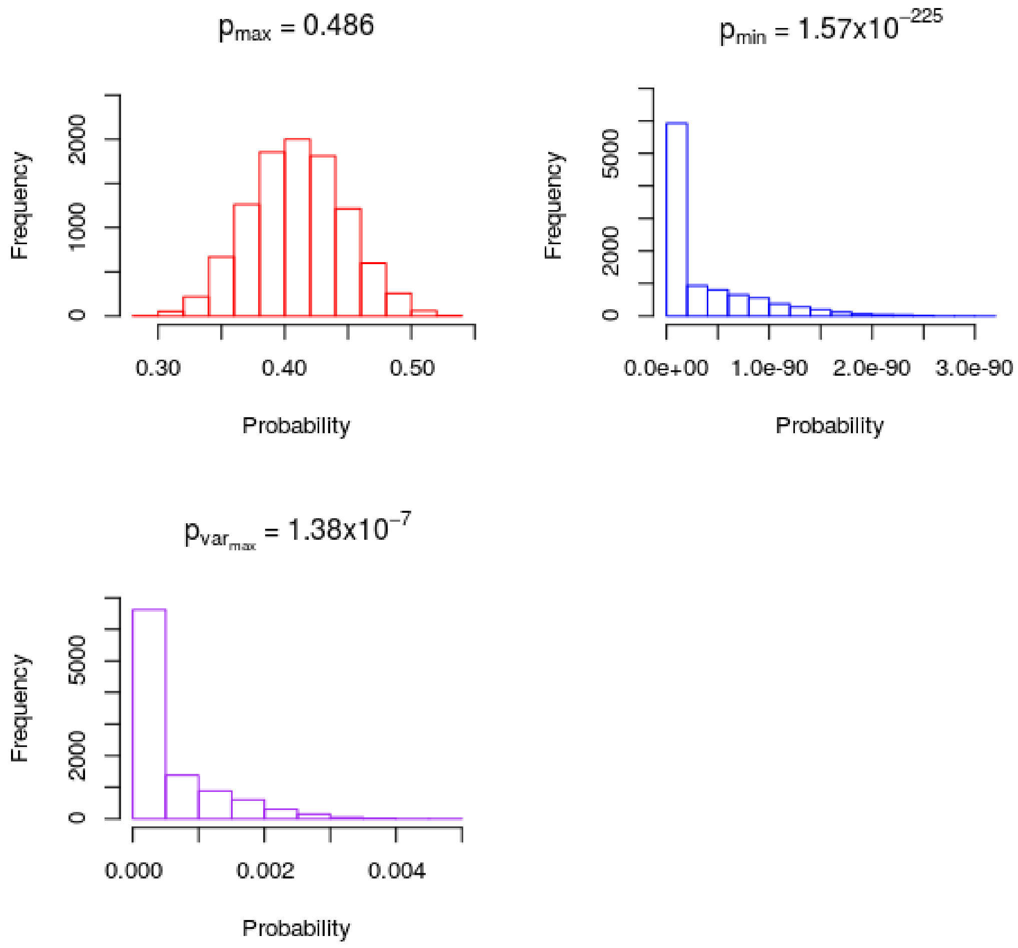

Figure S15: Distributions of the shuffled versions of the maximum probability distribution (red), the minimum positive probability (blue), and the probability corresponding to the maximum noise variance (purple).

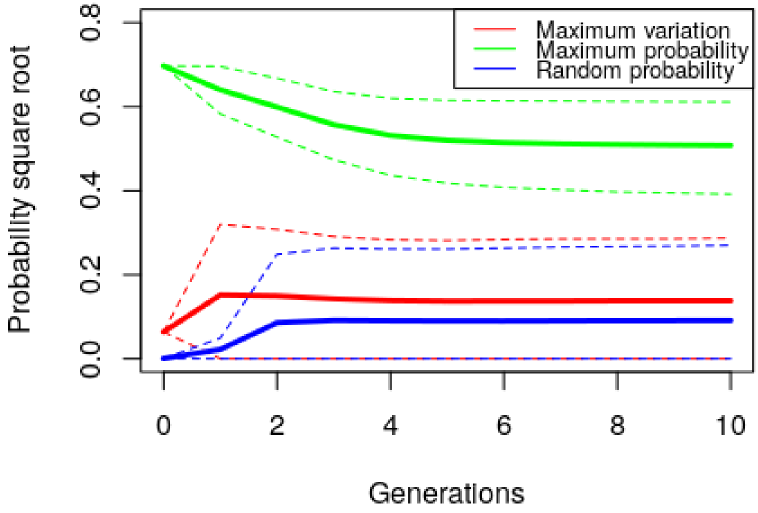

Figure S16: Evolution of the probabilities during 10 generations by applying the shuffling process. The red, green and blue lines show, respectively, the average evolution of the maximum probability, the minimum positive probability and the probability that has undergone the greatest variation. The dashed lines give the pointwise 95% confidence envelopes.

S2 Supporting text: Discussion on parameter estimation and model validation

In the perspective of fitting our demo-genetic model to data, a two-stage estimation approach (Pagan, 1986; Mrkvička et al., 2014; Dvořák et al., 2019) grounded on the conditional structure of our model (i.e., the genetics is modeled given the kinetics) could be explored: first, one may estimate parameters of the kinetic component of the model for each host separately to account for inter-host heterogeneity (using standard and validated kinetic models for the virus of interest); second, one may estimate parameters of the genetic component of the model conditional on fitted kinetics, assuming that genetic parameters take the same values for all the hosts (except if the amount of information contained in the data allows the estimation of genetic parameters at the host level).

The first stage could be implemented using a standard approach in viral kinetics. For instance, using diverse forms of mean-square error estimation, various kinetic models were fitted to daily viral load data (Baccam et al., 2006; Wang et al., 2020; Pinky and Dobrovolny, 2020; Hernandez-Vargas and Velasco-Hernandez, 2020), to CD8+ T-cells data (Blanco-Rodriguez et al., 2021) or to associations of viral load data and IFN fold change data (Pawelek et al., 2012). Using multiple series for fitting a model might allow the number of kinetic parameters and initial conditions that are preliminary fixed (which is a common practice in viral-kinetics modeling) to be reduced.

The second stage could lie on serial samples collected from each individual and revealing within-host evolution of the pathogen population, typically obtained from whole genome high-throughput sequencing (HTS) or high-throughput amplicon sequencing (HTAS). After the transformation of data in summary statistics, one might fit the genetic component of the model with approximate Bayesian computation (ABC; Beaumont, 2010; Csilléry et al., 2010; Marin et al., 2012). The summary statistics could be some diversity curves such as those presented in this article, or at least some points along these curves depending on HTS or HTAS data frequency in time. Since the summary statistics will contain some temporal dependencies, one might apply an ABC technique for weighting them and hence mitigating the effect of redundant summary statistics while reducing the number of model simulations (e.g., Soubeyrand et al., 2013). Using the Bayesian paradigm offers the possibility to circumvent possible parameter-identifiability issues since the posterior distribution of parameters provided by ABC could reveal the 'equivalence' of different parameter vectors in their ability to 'explain' the observed data. In addition, ABC offers an easy way to take into account the uncertainty of kinetic parameters used as input of the genetic component of the model. Indeed, in the fit of kinetic models to data, some of

the authors mentioned above apply a bootstrap step to measure the uncertainty of parameter estimates. This uncertainty could be propagated in ABC by generating multiple likely kinetics for each host and simulating genetic dynamics conditional on each of these kinetics.

The ABC tool kit also provides techniques for model validation and model choice (Csilléry et al., 2010; Pudlo et al., 2016). Such techniques could be applied to measure the goodness-of-fit of the whole model structure and compare models with different components related to both kinetics and genetic. Ideally, data not used for fitting the model should be used for this task. In addition, a classic practice in ABC is to use, for validation and comparison of models, summary statistics that were not used for fitting the model. Thus, one might exploit original diversity curves (not too much correlated with diversity curves used for parameter estimation) and original temporal series related to the kinetics (e.g., IFN data if only viral load data were used for fitting the model).

References

Baccam, P., Beauchemin, C., Macken, C. A., Hayden, F. G., and Perelson, A. S. (2006). Kinetics of influenza A virus infection in humans. *Journal of Virology* 80, 7590–7599

Beaumont, M. A. (2010). Approximate Bayesian computation in evolution and ecology. *Annual Review of Ecology, Evolution, and Systematics* 41, 379–406

Blanco-Rodriguez, R., Du, X., and Hernandez-Vargas, E. (2021). Computational simulations to dissect the cell immune response dynamics for severe and critical cases of SARS-CoV-2 infection. *Computer Methods and Programs in Biomedicine* 211, 106412

Csilléry, K., Blum, M. G., Gaggiotti, O. E., and François, O. (2010). Approximate Bayesian computation (ABC) in practice. *Trends in Ecology & Evolution* 25, 410–418

Dvořák, J., Møller, J., Mrkvička, T., and Soubeyrand, S. (2019). Quick inference for log Gaussian Cox processes with non-stationary underlying random fields. *Spatial Statistics* 33, 100388

Hernandez-Vargas, E. A. and Velasco-Hernandez, J. X. (2020). In-host mathematical modelling of COVID-19 in humans. *Annual Reviews in Control* 50, 448–456

Marin, J.-M., Pudlo, P., Robert, C. P., and Ryder, R. J. (2012). Approximate Bayesian computational methods. *Statistics and Computing* 22, 1167–1180

Mrkvička, T., Muška, M., and Kubečka, J. (2014). Two step estimation for Neyman-Scott point process with inhomogeneous cluster centers. *Statistics and Computing* 24, 91–100

Pagan, A. (1986). Two stage and related estimators and their applications. *The Review of Economic Studies* 53, 517–538

Pawelek, K. A., Huynh, G. T., Quinlivan, M., Cullinane, A., Rong, L., and Perelson, A. S. (2012). Modeling within-host dynamics of influenza virus infection including immune responses. *PLoS Computational Biology* 8, e1002588

Pinky, L. and Dobrovolny, H. M. (2020). SARS-CoV-2 coinfections: Could influenza and the common cold be beneficial? *Journal of Medical Virology* 92, 2623–2630

Pudlo, P., Marin, J.-M., Estoup, A., Cornuet, J.-M., Gautier, M., and Robert, C. P. (2016). Reliable ABC model choice via random forests. *Bioinformatics* 32, 859–866

Soubeyrand, S., Carpentier, F., Guiton, F., and Klein, E. K. (2013). Approximate Bayesian computation with functional statistics. *Statistical Applications in Genetics and Molecular Biology* 12, 17–37

Wang, S., Pan, Y., Wang, Q., Miao, H., Brown, A. N., and Rong, L. (2020). Modeling the viral dynamics of SARS-CoV-2 infection. *Mathematical Biosciences* 328, 108438
